# Regional Variations and Inequalities in Testing for Early Detection of Breast and Cervical Cancer: Evidence From a Nationally Representative Survey in India

**DOI:** 10.2188/jea.JE20240065

**Published:** 2025-03-05

**Authors:** Md. Mahfuzur Rahman, Md. Shafiur Rahman, Md. Rashedul Islam, Stuart Gilmour, Rei Haruyama, Atul Budukh, Abhishek Shankar, Gauravi Mishra, Ravi Mehrotra, Tomohiro Matsuda, Manami Inoue, Sarah Krull Abe

**Affiliations:** 1 Graduate School of Public Health, St. Luke’s International University, Tokyo, Japan; 2 Division of Prevention, National Cancer Center Institute for Cancer Control, Tokyo, Japan; 3 School of Health Innovation, Kanagawa University of Human Services, Kanagawa, Japan; 4 Hitotsubashi Institute for Advance Study, Hitotsubashi University, Tokyo, Japan; 5 Bureau of International Health Cooperation, National Center for Global Health and Medicine, Tokyo, Japan; 6 Tata Memorial Centre, Homi Bhabha National Institute (HBNI), Training School Complex, Anushakti Nagar, Mumbai, India; 7 Department of Radiation Oncology, Dr BR Ambedkar Institute Rotary Cancer Hospital, All India Institute of Medical Sciences, Delhi, India; 8 Department of Preventive Oncology, Tata Memorial Hospital, Mumbai, India; 9 Centre for Health, Innovation and Policy Foundation, Noida, India

**Keywords:** breast cancer, early cancer detection, cervical cancer, inequality, India, NFHS

## Abstract

**Background:**

The burden of cancer in India has been rising, yet testing for early detection remains low. This study explored inequalities in the uptake of breast cancer (BC) examination and cervical cancer (CC) among Indian women, focusing on socioeconomic, regional, and educational differences.

**Methods:**

Data from the 2019–21 National Family Health Survey (*n* = 353,518) were used to assess the uptake of BC examination and CC testing. Inequalities were quantified using the slope index of inequality (SII), relative index of inequality (RII), and relative concentration index (RCI). SII measured absolute inequality, while RII and RCI assessed relative inequality between disadvantaged and advantaged groups.

**Results:**

The ever uptake of tests for early detection of BC and CC were low at 9 and 20 per 1,000 women, respectively. Higher uptake was observed among women from the richest households compared to the poorest (SII: 1.1 for BC and 1.8 for CC). The magnitude of relative socioeconomic inequalities was more pronounced in rural areas (RCI: 22.5 for BC and 21.3 for CC) compared to urban areas. Similarly, higher-educated women were 4.84 times (RII: 4.84) and 2.12 times (RII: 2.12) more likely to undergo BC examination and CC testing, respectively, compared to non-educated women. The Northeastern region exhibited greater socioeconomic inequality, while the Western region showed more education-based inequality.

**Conclusion:**

The lower uptake of BC examination and CC testing and the marked inequalities underscore the need for targeted interventions to improve access and utilization of testing services, especially among lower-educated women, and those in rural areas.

## INTRODUCTION

Globally cancer causes a significant burden of disease, and this burden is projected to continue rising for at least the next 2 decades.^1^^–^^4^ Despite the potential for minimizing the long-term burden of breast cancer (BC) and cervical cancer (CC) through early detection and timely treatment, screening programs are limited in many low- and middle-income countries (LMIC), like India.^5^^,^^6^ In India, among all cancers, BC had the highest incidence and mortality rates among women in 2020 (age-standardized incidence rate of 24.2 per 100,000 and mortality rate of 11.7 per 100,000), followed by CC (incidence rate of 16.8 per 100,000 and mortality rate of 10.2 per 100,000).^4^^,^^7^ There is an increasing trend of BC incidence and mortality across India.^8^^–^^11^ This high burden of BC and CC often leads families to become impoverished due to resource-intensive treatment.^12^

Early detection, either through screening of asymptomatic individuals in a defined population or early diagnosis of symptomatic individuals, and timely treatment are crucial for minimizing the long-term burden of disease and death associated with BC and CC.^13^ Furthermore, early detection can reduce the treatment cost.^14^ However, the uptake of tests for early cancer detection in India is very low (around 1 in 100 women).^15^^,^^16^ Previous studies have reported significant inequalities in breast examination based on factors such as economic background, marital status, place of residence, and religion.^17^^–^^21^ These socioeconomic inequalities in the uptake of cancer screening also vary across different regions of the country. Although there is a study which investigated the level of socioeconomic inequality in the screening of BC and CC in India,^22^ no study has yet explored education-based inequality in the uptake of early detection of BC and CC at the national and sub-national levels. Reducing the inequality in access to screening tests is an important factor for early detection and improved outcome in BC and CC.

The uptake of cancer screening in LMICs, including India, is hindered by various factors, such as no or low awareness regarding cancer prevention and screening among women, inadequate health education, and financial and practical barriers to screening access.^20^^,^^21^ There are studies focused on the association between individual-level factors and CC screening,^21^ but none of them took community-level factors into account. It is important to identify barriers at individual, household, and community levels to improve the uptake of tests for early detection and ensure appropriate treatment. This study investigates the socioeconomic, regional, and education-based variations in the uptake of tests for early detection of BC and CC among Indian women. Additionally, we aimed to identify potential factors that influence the uptake of BC examination and CC testing.

## METHODS

### Study design and setting

Data from the most recent National Family Health Survey conducted in India during 2019–21 (NFHS-5), the fifth in the series, were used. The NHFS-5 collected information on health, nutrition and family welfare-related indicators in each state and union territory (UT) of India. The NFHS-5 used multi-stage stratified sampling to cover 707 districts, 28 states, and 8 union territories. The 2011 census provided the sampling frame for primary sampling unit (PSU) selection. In rural areas, PSUs were considered to be villages, while Census Enumeration Blocks (CEBs) were used in urban areas. The final sample PSUs were selected using probability proportional to size systematic sampling. The sampling weights in the NHFS-5 were estimated by the survey authority based on the sampling probabilities for each stage and each cluster separately. Details of sampling procedure and sampling weight calculations are presented elsewhere.^23^^,^^24^ Out of the 664,972 households selected from 30,198 PSUs, interviews were completed in 636,699 households, with a response rate of 97%. Among these households, a total of 747,176 women aged 15–49 years were identified, and interviews were completed with 724,115 women, with a response rate of 97% (eFigure 1).

### Study participants

The NFHS-5 interviewed 724,115 women aged 15–49 years. According to the cancer screening guidelines in India,^25^ women aged 30 years and over should get screened for CC using visual inspection with acetic acid, and BC using clinical breast examination. Thus, the participants in this study were limited to women aged 30–49 years. After excluding participants with missing information on key exposures, covariates, or outcomes, the analytical sample size for this study became 353,518 women aged 30–49 years (eFigure 1).

### Outcomes

The outcomes of this study were self-reported lifetime experience of breast examination and CC tests. To assess the lifetime uptake of BC examination and CC testing, the respondents were asked whether they had ever undergone a breast examination for breast cancer and a screening test for cervical cancer. The response to both questions was recorded as “yes” or “no”.

### Key exposures and covariates

Independent variables were chosen to represent demographics, health behavior, and community-level characteristics.^26^ Accordingly, we have included individual-, household- and community-level variables as independent variables. Details of the covariates including estimation methods are presented in eTable 1. The demographic characteristics include women’s age (30–34 years, 35–39 years, 40–44 years, 45–49 years), marital status (currently married, others [divorced/separated]), education level of the respondent (no education, primary education, secondary education, higher education), number of children ever born (none, 1–2, and 3 or more), sex of the household head (male, female), wealth quintile (a proxy measure of household socioeconomic status [SES]; poorest, poorer, middle class, richer, richest), health insurance (no, yes), and religion (Hindu, Muslim, Christian, Others). Health behavior factors include drinking habits (no, yes), tobacco consumption (no, yes), body mass index (BMI) (underweight [BMI <18.5 kg/m^2^], normal [BMI between 18.5 to <25 kg/m^2^], overweight or obese [BMI >25.0 kg/m^2^]), and exposure to mass media (no access, minimum access, moderate access, and higher access). The community level characteristics were broken down into community education level (low, high), place of residence (urban, rural), region (North, Central, East, Northeast, West, South), state, and union territory levels.

### Statistical analysis

The uptake of tests for early detection were presented as rates per 1,000 women. Frequency distributions and univariate analysis were used to describe background characteristics. Chi-square tests were performed to quantify the significant difference in the proportion of tests for early cancer detection between different categories of covariates. All statistical analysis accounted for sampling weights. Both absolute and relative measures of inequality were used to measure the level and extent of inequality based on household socioeconomic status and respondents’ level of education. A regression-based slope index of inequality (SII) was used as an absolute measure of inequality between the disadvantaged and advantaged sub-groups.^27^ A positive value of SII indicates that the indicator is more prevalent in the advantaged subgroup than their counterparts. On the other hand, the relative index of inequality (RII) and relative concentration index were used to assess the magnitude and extent of relative inequality. The RII is a statistical measure that quantifies the level of inequality by comparing the estimated indicator values of the most advantaged subgroup to those of the most disadvantaged subgroup, while also considering the circumstances in all other subgroups.^27^ The allocation of weights to subgroups is determined based on their proportionate representation within the overall population. RII produces only positive values; when the value of RII is 1, there is no inequality; values greater than one indicate that the advantaged group is more likely to have the outcome, and vice versa for values less than one. The relative concentration index (RCI) was used to measure the concentration of the indicator between advantaged and disadvantaged subgroups on a relative scale. The RCI values range between −1 and 1. For simplicity, we multiplied this value by 100. The zero value of RCI indicates there is no inequality, a positive value of RCI indicates the indicators are more concentrated among the advantaged subgroups, and a negative value the opposite. In addition, mixed-effects Poisson regression models with a random intercept at individual-, household-, and community-level factors were used to identify potential risk factors associated with the uptake of tests for early cancer diagnosis. Since the proportion of the outcome was low, a modified Poisson regression was used with a robust error variance.^28^ Both crude model and adjusted models were used to estimate prevalence ratio (PR) and 95% confidence intervals (CI). Model 1 was the unadjusted model, model 2 was adjusted for demographic and health behavior factors, and model 3 was further adjusted for community level-factors. Data management and analysis were performed in Stata MP version 17 (Stata Corp, College Station, TX, USA).

## RESULTS

### Background characteristics

Table 1 represents the background characteristics of the study population. Overall, a total of 353,518 Indian women aged 30–49 years were included in this study. About one-third of the included women (35.5%) had no education and 10.3% had a higher education. Approximately 91% of women were currently married and one-third were overweight (34.2%). More than 80% of the participants were Hindu, while 12.1% and 2.6% were Muslim and Christian, respectively. Women headed 16.5% of participants’ households and two-thirds lived in rural areas (66.4%).

**Table 1.** Background characteristics of included Indian women

Characteristics	Number of participants (%)	Uptake per 1,000 women (95% CI)	

		Breast cancer examination	Cervical cancer test
Overall	353,518	8.8 (8.5–9.1)	19.8 (19.3–20.3)
***Individual-level characteristics***			
Age group, years			
30–34	98,041 (27.5)	7.6 (7.1–8.2)	15.9 (15.1–16.7)
35–39	95,023 (26.7)	8.4 (7.9–9.1)	18.9 (18.0–19.7)
40–44	78,915 (22.4)	10.1 (9.4–10.8)	21.6 (20.6–22.7)
45–49	81,539 (23.4)	9.3 (8.7–10.0)	23.7 (22.7–24.8)
Highest educational level^a^			
No education	128,728 (35.5)	4.4 (4.1–4.8)	14.3 (13.7–15.0)
Primary education	54,768 (15.5)	8.8 (8.1–9.6)	21.0 (19.8–22.2)
Secondary education	138,391 (38.7)	10.9 (10.4–11.5)	23.0 (22.2–23.8)
Higher education	31,631 (10.3)	15.9 (14.7–17.3)	25.0 (23.4–26.6)
Marital status			
Currently married	320,627 (91.1)	8.8 (8.5–9.1)	19.9 (19.4–20.3)
Others (divorced/separated)	32,891 (8.9)	8.5 (7.6–9.6)	19.3 (17.8–20.9)
Body mass index			
Underweight (BMI <18.5 kg/m^2^)	36,856 (10.4)	6.6 (5.8–7.5)	15.6 (14.4–17.0)
Normal (BMI 18.5 to <25 kg/m^2^)	204,331 (55.4)	6.6 (6.3–7.0)	16.1 (15.6–16.7)
Overweight or Obese (BMI >25.0 kg/m^2^)	112,331 (34.2)	13.0 (12.3–13.6)	27.0 (26.1–27.9)
Exposure to mass-media^b^			
No access	93,651 (24.8)	3.6 (3.2–4.0)	11.7 (11.0–12.4)
Minimum access	54,025 (14.1)	5.7 (5.1–6.4)	16.7 (15.6–17.9)
Moderate access	161,053 (46.8)	10.9 (10.4–11.5)	23.4 (22.7–24.1)
Higher access	44,789 (14.3)	13.8 (12.9–14.9)	25.2 (23.9–26.6)
Number of children ever born			
None	17,847 (4.6)	9.2 (7.8–10.8)	20.3 (18.3–22.6)
1–2	159,581 (47.8)	11.7 (11.2–12.2)	23.6 (22.9–24.3)
3 or more	176,090 (47.6)	5.8 (5.5–6.2)	16.0 (15.4–16.6)
Drinks alcohol^c^			
No	343,871 (98.9)	8.9 (8.6–9.2)	19.9 (19.4–20.3)
Yes	9,647 (1.1)	1.6 (0.7–3.5)	12.9 (9.8–17.1)
Tobacco use^d^			
No	320,968 (93.8)	9.1 (8.8–9.4)	20.4 (19.9–20.9)
Yes	32,550 (6.2)	4.1 (3.3–5.0)	10.8 (9.5–12.2)
Religion			
Hindu	268,524 (82.4)	9.1 (8.7–9.4)	20.2 (19.7–20.7)
Muslim	39,758 (12.1)	5.2 (4.6–6.0)	11.7 (10.7–12.8)
Christian	26,765 (2.6)	13.5 (11.3–16.1)	37.8 (34.1–42.0)
Other	18,471 (2.9)	11.1 (9.2–13.3)	26.3 (23.4–29.6)
Membership in health insurance scheme			
No	224,780 (65.5)	8.9 (8.6–9.3)	18.9 (18.4–19.5)
Yes	128,738 (34.5)	8.5 (8.0–9.0)	21.5 (20.7–22.3)
***Household-level characteristics***			
Household socioeconomic status^e^			
Poorest	71,508 (17.9)	3.7 (3.3–4.2)	9.9 (9.1–10.7)
Poorer	75,870 (19.2)	6.7 (6.1–7.4)	16.3 (15.3–17.3)
Middle	73,935 (20.6)	9.1 (8.5–9.8)	22.4 (21.3–23.5)
Richer	69,035 (21.2)	10.0 (9.3–10.7)	23.9 (22.8–25.0)
Richest	63,170 (21.1)	13.5 (12.7–14.4)	24.8 (23.7–26.0)
Sex of household head			
Male	295,253 (83.5)	8.6 (8.3–9.0)	19.7 (19.2–20.2)
Female	58,265 (16.5)	9.7 (8.9–10.5)	20.4 (19.3–21.6)
***Community-level characteristics***			
Place of residence			
Urban areas	90,978 (33.6)	12.8 (12.1–13.4)	23.9 (23.0–24.8)
Rural areas	262,540 (66.4)	6.8 (6.5–7.1)	17.7 (17.2–18.3)
Region			
North	71,128 (13.8)	2.5 (2.1–3.0)	9.1 (8.2–9.9)
Central	74,908 (22.3)	4.0 (3.6–4.5)	12.8 (12.0–13.6)
East	55,138 (22.0)	2.2 (1.9–2.5)	5.6 (5.1–6.2)
Northeast	52,636 (3.8)	3.5 (2.7–4.7)	5.6 (4.4–7.0)
West	37,498 (14.8)	9.7 (8.9–10.5)	17.1 (16.0–18.3)
South	62,210 (23.2)	23.7 (22.7–24.8)	50.4 (48.9–51.9)
Community education^f^			
Low	110,648 (30.1)	3.4 (3.0–3.7)	11.6 (10.9–12.2)
High	242,870 (69.9)	11.1 (10.7–11.5)	23.3 (22.7–23.9)

BMI, body mass index; CI, confidence interval.

Data Source: National Family Health Survey (NFHS-5), 2019–21.

^a^Respondents’ education level was classified as no education (0 years of schooling), primary education (1–5 years of schooling), secondary education (6–12 years of schooling), and higher education (13 or more years of schooling).

^b^Exposure to mass media—a composite variable—was generated based on the respondent’s self-reported information on the frequency of reading newspaper or magazine, listening radio and watching television. The response options for these exposures to mass-media were “not at all” (assigned the value 0), “less than once a week” (assigned the value 1), and “at least once a week” (assigned the value 2). Then the value of these questions was summed to get a composite value. The composite value ranged from 0 to 6 and categorized as “no access” for the value of 0, “minimum access” for the value of 1, “moderate access” for the value between 2–3, and “high access” for the value between 4–6.

^c^Alcohol use was defined as the current use of alcohol.

^d^Tobacco use was defined as the current use of tobacco in any form (both smoking and smokeless tobacco products were included).

^e^Household socioeconomic status was measured using the wealth score. The households were ranked based on these scores and subsequently divided into quintiles, each representing 20% of the population. These quintiles were categorized as follows: poorest (lowest 20%), poorer, middle, richer, and richest (highest 20%).

^f^Community-level education was calculated on the basis of women’s education level in the community and the national aggregate. From the survey results, the number of years of formal education was derived for each woman. The national aggregate was determined by calculating the median years of formal education for all women included in the sample. According to the NFHS-5, the median duration of female schooling was reported to be 5 years. Clusters were categorized as having either lower or higher levels of female education based on whether their median level of female education was lower or higher than the national average, respectively.

### Uptake of BC examination and CC testing

**Figure 1.**
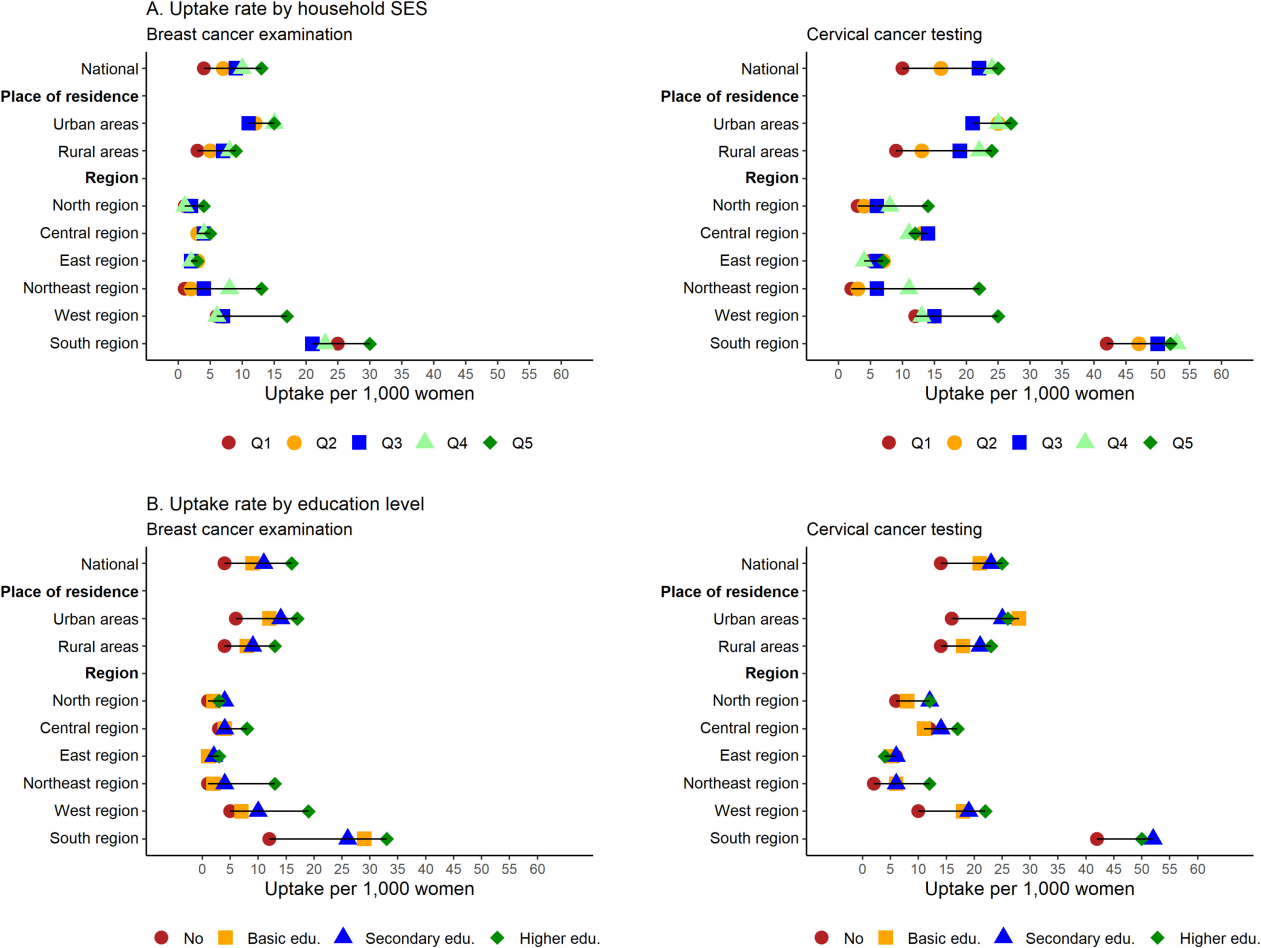
Uptake of breast cancer examination and cervical cancer test among Indian women by household socioeconomic status and level of education at national and sub-national level. Panel A presents the uptake of breast cancer examination and cervical cancer testing by household socio-economic status and panel B presents the uptake of breast cancer examination and cervical cancer testing by women’s level of education. Exact values with 95% confidence intervals are presented in eTable 5 and eTable 6. Household socioeconomic status was measured using wealth scores. The households were ranked based on these scores and subsequently divided into quintiles, each representing 20% of the population. These quintiles were categorized as follows: poorest (lowest 20%), poorer, middle, richer, and richest (highest 20%). Respondents’ education level was classified as no education (0 years of schooling), primary education (1–5 years of schooling), secondary education (6–12 years of schooling), and higher education (13 or more years of schooling). Q1, poorest quintile; Q2, poorer quintile; Q3, middle quintile; Q4, richer quintile; Q5, richest quintile; SES, socioeconomic status.

Overall, the ever uptake of BC examination and CC testing were 8.8 and 19.8 per 1,000 women, respectively (Table 1). The uptake rate (per 1,000 women) was higher among higher-educated women (15.9 for BC; 25.0 for CC) and among those from rich households (13.5 for BC; 24.8 for CC) (Figure 1). Regional variation in the uptake of tests for early cancer detection was observed; uptake rates were highest in the Southern region (23.7 for BC and 50.4 for CC), while the rates were lower in the Eastern and Northern regions (Table 1, Figure 1). At state level, the highest uptake rate was observed in Tamil Nadu (58.3 for BC; 101.3 for CC), followed by Mizoram and Kerala state (eTable 2). Puducherry had the highest uptake rate among the union territories (41.6 for BC; 74.6 for CC). The uptake of BC examination and CC testing by household SES and education at state and union territory levels are presented in eTable 5, eTable 6, eFigure 2, and eFigure 3. When household SES and education were taken together, a higher uptake rate was observed among educated women from affluent households (eFigure 4).

### Socioeconomic inequality in the uptake of BC examination and CC testing

**Figure 2.**
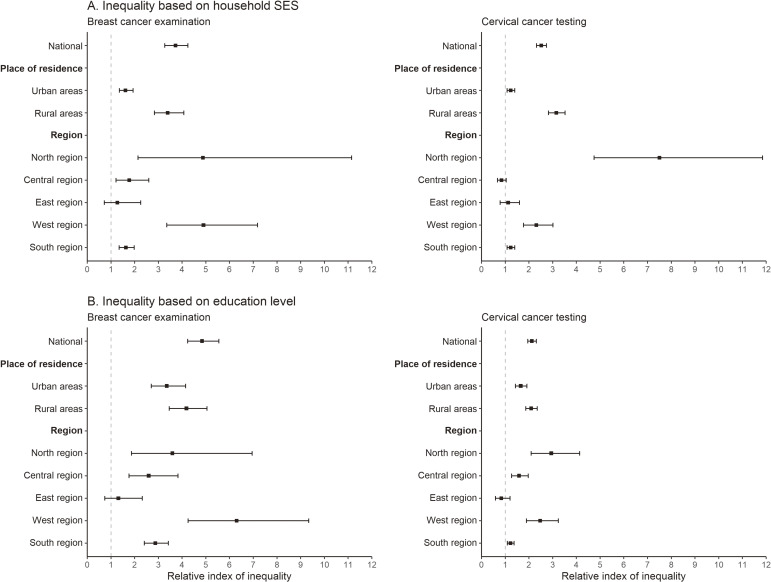
Relative inequality in the uptake of breast cancer examination and cervical cancer test among Indian women at the national and sub-national levels. Panel A presents the values of relative inequality (relative index of inequality) of breast cancer examination and cervical cancer testing by socio-economic status and panel B presents the values of relative inequality of breast cancer examination and cervical cancer testing by women’s level of education Exact values with 95% confidence intervals are presented in eTable 9. The model did not converge for the Northeastern region. Thus, it was excluded from this analysis. Household socioeconomic status was measured using wealth scores. The households were ranked based on these scores and subsequently divided into quintiles, each representing 20% of the population. These quintiles were categorized as follows: poorest (lowest 20%), poorer, middle, richer, and richest (highest 20%). Respondents’ education level was classified as no education (0 years of schooling), primary education (1–5 years of schooling), secondary education (6–12 years of schooling), and higher education (13 or more years of schooling); SES, socioeconomic status.

The magnitude of absolute and relative socioeconomic inequalities in the uptake of tests for early cancer detection are presented in Table 2 and Figure 2. The positive values of SII indicate that the ever uptake of BC examination and CC testing was higher among women from the richest households compared to the poorest households, confirming pro-rich inequality in early cancer detection at the national level (SII: 1.1 for BC and 1.8 for CC). Similarly, the magnitude of relative socioeconomic inequality was found to be large at the national level (RCI: 24.4 for BC and 20.3 for CC). The magnitude of relative socioeconomic inequality in the uptake of both BC examination and CC testing was found to be higher among women from rural areas (RCI: 22.5 and 21.3 respectively) compared to urban areas (RCI: 7.3 and 5.8 respectively). At the regional level, the magnitude of socioeconomic inequality in the uptake of BC examination was highest in the Northeastern region (RCI: 32.8) followed by the Western region (RCI: 18.2). For CC testing, the magnitude of pro-rich relative inequality was highest in the Northeastern region (RCI: 36.6), followed by the Northern (RCI: 25.1), and Eastern region (RCI: 9.0). The state-specific analysis revealed that women from richest households in the Western region were 4.9 times (RII: 4.9) more likely to undergo BC examination compared to women from poorest households (Figure 2). At the state level, the magnitude of pro-rich inequality in CC testing was found to be very high in Uttarakhand (RCI: 42.5), followed by Mizoram (RCI: 32.4), Jharkhand (RCI: 27.5), and Gujarat state (RCI: 20.9), while pro-rich relative inequality in BC examination was observed in Manipur (RCI: 32.2), followed by Assam (RCI: 30.8) (eTable 7).

**Table 2.** Socioeconomic and education-based absolute and relative inequalities in uptake of breast cancer examination and cervical cancer test among Indian women

	Breast cancer examination		Cervical cancer test	
	
	SII (95% CI)	RCI (95% CI)	SII (95% CI)	RCI (95% CI)
***Socioeconomic inequality*** *^a^*				
National	1.1 (0.9–1.3)^†^	24.4 (21.7–27.0)^†^	1.8 (1.6 to 2.1)^†^	20.3 (18.3–22.3)^†^
Place of residence				
Urban areas	0.6 (0.2–1.0)^§^	7.3 (2.6–12.0)^§^	0.5 (−0.1 to 1.0)	5.8 (2.1–9.4)^§^
Rural areas	0.8 (0.6–1.0)^†^	22.5 (19.2–25.7)^†^	2.0 (1.7–2.3)^†^	21.3 (18.9–23.7)^†^
Region				
North	0.3 (0.2–0.5)^†^	12.3 (3.3–21.3)^§^	1.5 (1.2–1.9)^†^	25.1 (18.8–31.4)^†^
Central	0.2 (0.04–0.4)^*^	9.7 (1.2–18.2)^*^	−0.2 (−0.6 to 0.1)	−5.7 (−10.7 to −0.7)^*^
East	0.1 (−0.1 to 0.2)	3.9 (−7.5 to 15.2)	0.1 (−0.2 to 0.3)	9.0 (2.7–15.4)^§^
Northeast	0.9 (0.6–1.2)^†^	32.8 (25.0–40.7)^†^	1.4 (1.1–1.7)^†^	36.6 (30.7–42.5)^†^
West	1.4 (0.6–2.2)^§^	18.2 (8.2–28.3)^†^	1.3 (0.4–2.3)^§^	8.3 (1.4–15.2)^*^
South	1.0 (0.4–1.6)^§^	9.1 (4.8–13.3)^†^	0.8 (0.0–1.6)^*^	5.4 (2.6–8.3)^†^
***Education-based inequality*** *^b^*				
National	1.4 (1.2–1.6)^†^	30.8 (28.3–33.3)^†^	1.5 (1.2–1.7)^†^	17.8 (15.9–19.7)^†^
Place of residence				
Urban areas	1.3 (0.9–1.7)^†^	23.6 (20.0–27.1)^†^	1.0 (0.4–1.5)^†^	13.6 (10.6–16.7)^†^
Rural areas	0.9 (0.8–1.1)^†^	27.2 (23.7–30.8)^†^	1.3 (1.0–1.6)^†^	15.4 (13.1–17.8)^†^
Region				
North	0.3 (0.1–0.5)^†^	8.6 (−0.8 to 18.1)	1.0 (0.6–1.3)^†^	14.5 (9.1–19.8)^†^
Central	0.4 (0.2–0.6)^§^	11.9 (3.8–20.0)^§^	0.6 (0.2–1.0)^§^	0.6 (−4.2 to 5.3)
East	0.1 (−0.1 to 0.2)	3.0 (−9.2 to 15.2)	−0.1 (−0.4 to 0.2)	6.1 (−1.1 to 13.3)
Northeast	1.0 (0.6–1.3)^†^	23.9 (16.7–31.2)^†^	0.9 (0.6–1.2)^†^	12.0 (7.3–16.7)^†^
West	1.6 (0.8–2.4)^†^	30.9 (22.8–39.1)^†^	1.4 (0.5–2.3)^§^	17.3 (10.9–23.6)^†^
South	2.2 (1.6–2.8)^†^	23.2 (20.0–26.3)^†^	0.8 (0.0–1.6)	8.7 (5.9–11.5)^†^

CI, confidence interval; RCI, relative concentration index; SII, Slope index of inequality.

^*^P < 0.05; ^§^*P* < 0.01; ^†^*P* < 0.001.

^a^Household socioeconomic status was measured using the wealth score. The households were ranked based on these scores and subsequently divided into quintiles, each representing 20% of the population. These quintiles were categorized as follows: poorest (lowest 20%), poorer, middle, richer, and richest (highest 20%).

^b^Respondents’ education level was classified as no education (0 years of schooling), primary education (1–5 years of schooling), secondary education (6–12 years of schooling), and higher education (13 or more years of schooling).

### Education-based inequality in the uptake of BC examination and CC testing

A higher magnitude of education-based relative inequality was observed for BC examination (RCI: 30.8) and CC testing (RCI: 17.8), indicating that the ever uptake of tests for early detection was higher among higher-educated women compared to non-educated counterparts at the national level (Table 2). Higher-educated urban women were 3.35 times (RII: 3.35) and 1.65 times (RII: 1.65) more likely to undergo BC examination and CC testing, respectively, compared to non-educated women (Figure 2). At regional level, the magnitude of education-based inequality in the uptake of BC examination was highest in the Western region (RCI: 30.9), followed by Northeastern (RCI: 23.9), and Southern regions (RCI: 23.2) (Table 2). Similarly, the magnitude of education-based relative inequality in the uptake of the CC test was found to be highest in the Western region (RCI: 17.3), followed by the Northern region (RCI: 14.5). The magnitude of absolute and relative education-based inequalities in the uptake of BC examination and CC test at the state level are presented in eTable 8.

### Factors associated with the uptake of BC examination and CC testing

Table 3 represents the results of the mixed-effect modified Poisson regression models for the ever uptake of BC examination and CC testing. In addition to higher education and affluent households, advanced age, greater BMI, and Christian religion were associated with a higher uptake rate. Overweight women were more likely to undergo tests for early cancer detection (PR 1.27; 95% CI, 1.16–1.39 for BC and PR 1.21; 95% CI, 1.14–1.27 for CC) compared to women with a normal weight. In the case of religion, Christian women were more likely to undergo CC testing (PR 1.31; 95% CI, 1.17–1.47) than Hindu women, while Muslim women were less likely to undergo a test for BC (PR 0.80; 95% CI, 0.68–0.95) and CC (PR 0.79; 95% CI, 0.70–0.88). Regarding contextual factors, urban residence, higher levels of community education, and residence in the Southern region were associated with a higher uptake of tests for early cancer detection. Of note, women from the Southern region were more likely to undergo BC examination and CC testing than women from other regions. In addition, women from a community with higher average education were more likely to undergo tests for BC (PR 1.74; 95% CI, 1.47–2.06) and CC (PR 1.30; 95% CI, 1.16–1.45) compared to women from a community with relatively lower level of education.

**Table 3.** Factors associated with the uptake of breast cancer examination and cervical cancer test among Indian women

Characteristics	Prevalence ratio (PR) (95% confidence intervals)^a^					

	Breast cancer examination			Cervical cancer test		
	
	Model 1^b^	Model 2^c^	Model 3^d^	Model 1^b^	Model 2^c^	Model 3^d^
*Individual-level*						
Age group (years)						
30–34 (Ref.)	1.00	1.00	1.00	1.00	1.00	1.00
35–39	1.13 (1.00–1.27)^*^	1.15 (1.02–1.29)^*^	1.10 (0.98–1.23)	1.19 (1.10–1.28)^†^	1.18 (1.10–1.27)^†^	1.15 (1.07–1.24)^†^
40–44	1.37 (1.22–1.54)^†^	1.43 (1.28–1.61)^†^	1.34 (1.19–1.50)^†^	1.42 (1.32–1.54)^†^	1.39 (1.29–1.50)^†^	1.34 (1.24–1.44)^†^
45–49	1.29 (1.15–1.45)^†^	1.47 (1.29–1.66)^†^	1.33 (1.17–1.50)^†^	1.56 (1.45–1.69)^†^	1.56 (1.45–1.69)^†^	1.47 (1.36–1.59)^†^
Highest educational level^e^						
No education (Ref.)	1.00	1.00	1.00	1.00	1.00	1.00
Primary	1.65 (1.42–1.91)^†^	1.25 (1.08–1.45)^§^	1.18 (1.02–1.37)^*^	1.28 (1.17–1.39)^†^	1.07 (0.98–1.16)	1.08 (0.99–1.17)
Secondary	2.42 (2.17–2.70)^†^	1.53 (1.34–1.74)^†^	1.45 (1.27–1.65)^†^	1.52 (1.43–1.62)^†^	1.13 (1.05–1.22)^§^	1.16 (1.07–1.25)^†^
Higher	3.42 (2.98–3.93)^†^	1.77 (1.48–2.11)^†^	1.69 (1.41–2.03)^†^	1.76 (1.60–1.92)^†^	1.10 (0.98–1.23)	1.14 (1.02–1.27)^*^
Marital status						
Currently married	0.96 (0.84–1.11)	1.07 (0.90–1.26)	1.13 (0.96–1.34)	1.01 (0.93–1.11)	1.13 (1.02–1.26)^*^	1.18 (1.07–1.31)^§^
Widowed/divorced/others (Ref.)	1.00	1.00	1.00	1.00	1.00	1.00
Body mass index						
Underweight (BMI <18.5 kg/m^2^)	0.89 (0.75–1.05)	1.05 (0.89–1.23)	1.04 (0.89–1.22)	0.89 (0.81–0.99)^*^	0.98 (0.89–1.08)	0.97 (0.88–1.07)
Normal (BMI: 18.5 to <25 kg/m^2^)	1.00	1.00	1.00	1.00	1.00	1.00
Overweight or Obese (BMI >25.0 kg/m^2^)	1.91 (1.76–2.09)^†^	1.37 (1.25–1.51)^†^	1.27 (1.16–1.39)^†^	1.66 (1.57–1.75)^†^	1.27 (1.20–1.34)^†^	1.21 (1.14–1.27)^†^
Number of children ever born						
None	0.84 (0.70–1.01)	0.87 (0.71–1.06)	0.91 (0.74–1.10)	0.84 (0.74–0.94)^§^	0.95 (0.83–1.08)	0.98 (0.86–1.11)
1–2 (Ref.)	1.00	1.00	1.00	1.00	1.00	1.00
3 or more	0.57 (0.52–0.62)^†^	0.83 (0.76–0.91)^†^	0.99 (0.91–1.09)	0.71 (0.68–0.75)^†^	0.93 (0.88–0.98)^*^	1.05 (0.99–1.11)
Alcohol drinker^f^						
No (Ref.)	1.00	1.00	1.00	1.00	1.00	1.00
Yes	0.40 (0.27–0.60)^†^	0.53 (0.33–0.83)^§^	0.50 (0.31–0.78)^§^	0.7 (0.58–0.85)^†^	0.95 (0.77–1.16)	0.90 (0.73–1.10)
Tobacco user^g^						
No (Ref.)	1.00	1.00	1.00	1.00	1.00	1.00
Yes	0.96 (0.83–1.11)	1.14 (0.96–1.35)	1.26 (1.06–1.51)^§^	0.88 (0.80–0.97)^*^	1.05 (0.94–1.17)	1.20 (1.08–1.34)^§^
Membership in health insurance scheme						
No (Ref.)	1.00	1.00	1.00	1.00	1.00	1.00
Yes	0.88 (0.81–0.97)^§^	1.02 (0.93–1.13)	0.93 (0.85–1.03)	1.05 (0.99–1.10)	1.09 (1.02–1.15)^§^	0.99 (0.93–1.05)
Exposure to mass-media^h^						
No access (Ref.)	1.00	1.00	1.00	1.00	1.00	1.00
Minimum access	1.34 (1.13–1.59)^§^	1.05 (0.88–1.26)	0.94 (0.79–1.12)	1.27 (1.15–1.41)^†^	1.01 (0.92–1.12)	0.94 (0.86–1.04)
Moderate access	2.37 (2.09–2.70)^†^	1.32 (1.14–1.53)^†^	0.99 (0.86–1.15)	1.87 (1.74–2.01)^†^	1.14 (1.05–1.24)^§^	0.94 (0.87–1.02)
Higher access	3.81 (3.30–4.39)^†^	1.63 (1.36–1.95)^†^	1.14 (0.96–1.36)	2.35 (2.15–2.57)^†^	1.27 (1.15–1.42)^†^	0.99 (0.89–1.11)
*Household-level*						
Household socioeconomic status^i^						
Poorest	0.41 (0.35–0.48)^†^	0.77 (0.64–0.92)^§^	1.07 (0.89–1.29)	0.44 (0.40–0.49)^†^	0.69 (0.62–0.77)^†^	0.87 (0.78–0.97)^*^
Poorer	0.66 (0.58–0.75)^†^	0.87 (0.77–1.00)^*^	1.00 (0.88–1.15)	0.68 (0.63–0.74)^†^	0.84 (0.77–0.91)^†^	0.92 (0.85–1.00)^*^
Middle (Ref.)	1.00	1.00	1.00	1.00	1.00	1.00
Richer	1.06 (0.94–1.19)	0.93 (0.82–1.05)	0.89 (0.78–1.00)	1.06 (0.98–1.14)	0.98 (0.91–1.05)	0.96 (0.89–1.03)
Richest	1.27 (1.13–1.43)^†^	1.04 (0.91–1.19)	1.04 (0.90–1.20)	1.10 (1.02–1.19)^*^	1.08 (0.99–1.17)	1.10 (1.01–1.20)^*^
Male household head						
No (Ref.)	1.00	1.00	1.00	1.00	1.00	1.00
Yes	0.89 (0.80–0.99)^*^	0.93 (0.83–1.05)	0.99 (0.88–1.11)	0.96 (0.90–1.03)	0.97 (0.90–1.04)	1.01 (0.93–1.08)
Religion						
Hindu (Ref.)	1.00	1.00	1.00	1.00	1.00	1.00
Muslim	0.71 (0.61–0.83)^†^	0.80 (0.68–0.95)^*^	0.80 (0.68–0.95)^*^	0.64 (0.58–0.71)^†^	0.77 (0.68–0.86)^†^	0.79 (0.70–0.88)^†^
Christian	1.38 (1.20–1.58)^†^	1.15 (0.96–1.37)	1.08 (0.90–1.30)	1.31 (1.20–1.43)^†^	1.22 (1.09–1.37)^§^	1.31 (1.17–1.47)^†^
Other	0.66 (0.53–0.83)^†^	0.71 (0.56–0.90)^§^	1.13 (0.88–1.45)	1.01 (0.90–1.13)	0.91 (0.78–1.05)	1.36 (1.17–1.58)^†^
*Community-level*						
Place of residence						
Urban (Ref.)	1.00		1.00	1.00		1.00
Rural	0.50 (0.46–0.54)^†^		0.75 (0.65–0.86)^†^	0.65 (0.62–0.69)^†^		0.87 (0.78–0.97)^§^
Region						
North (Ref.)	1.00		1.00			1.00
Central	1.41 (1.16–1.70)^†^		1.60 (1.27–2.02)^†^	1.29 (1.16–1.43)^†^		1.74 (1.49–2.05)^†^
East	0.78 (0.62–0.99)^*^		1.06 (0.80–1.39)	0.67 (0.59–0.77)^†^		1.03 (0.85–1.25)
Northeast	1.72 (1.42–2.10)^†^		1.84 (1.43–2.37)^†^	1.10 (0.97–1.23)		1.29 (1.07–1.54)^§^
West	2.17 (1.78–2.66)^†^		1.70 (1.30–2.22)^†^	1.25 (1.10–1.41)^§^		1.50 (1.23–1.81)^†^
South	8.04 (6.88–9.41)^†^		8.27 (6.76–10.12)^†^	5.22 (4.79–5.69)^†^		7.74 (6.70–8.94)^†^
Community education^j^						
Low	1.00		1.00	1.00		1.00
High	2.86 (2.53–3.22)^†^		1.74 (1.47–2.06)^†^	1.70 (1.59–1.81)^†^		1.30 (1.16–1.45)^†^

BMI, body mass index; CI, confidence interval; Ref., reference category.

^*^P < 0.05; ^§^*P* < 0.01; ^†^*P* < 0.001.

^a^Mixed-effect Poisson regression model with a random intercept at household- and community-level were used to estimate prevalence ratio and 95% confidence intervals.

^b^Model 1: Unadjusted model.

^c^Model 2: Adjusted for individual and community-level factors (respondent’s age, level of educational, marital status, body mass index, number of children ever born, alcohol drinking habit, tobacco use, membership in health insurance scheme, exposure to mass-media, household socioeconomic status, sex of household head, and religion).

^d^Model 3: Further adjusted for contextual factors (place of residence, region, and level of education in the community).

^e^Respondents’ education level was classified as no education (0 years of schooling), primary education (1–5 years of schooling), secondary education (6–12 years of schooling), and higher education (13 or more years of schooling).

^f^Alcohol use was defined as the current use of alcohol.

^g^Tobacco use was defined as the current use of tobacco in any form (both smoking and smokeless tobacco products were included).

^h^Exposure to mass media—a composite variable—was generated based on the respondent’s self-reported information on the frequency of reading newspaper or magazine, listening radio and watching television. The response options for these exposures to mass-media were “not at all” (assigned the value 0), “less than once a week” (assigned the value 1), and “at least once a week” (assigned the value 2). Then the value of these questions was summed to get a composite value. The composite value ranged from 0 to 6 and categorized as “no access” for the value of 0, “minimum access” for the value of 1, “moderate access” for the value between 2–3, and “high access” for the value between 4–6.

^i^Household socioeconomic status was measured using the wealth scores. The households were ranked based on these scores and subsequently divided into quintiles, each representing 20% of the population. These quintiles were categorized as follows: poorest (lowest 20%), poorer, middle, richer, and richest (highest 20%).

^j^Community-level education was calculated on the basis of women’s education level in the community and the national aggregate. From the survey results, the number of years of formal education was derived for each woman. The national aggregate was determined by calculating the median years of formal education for all women included in the sample. According to the NFHS-5, the median duration of female schooling was reported to be 5 years. Clusters were categorized as having either lower or higher levels of female education based on whether their median level of female education was lower or higher than the national average, respectively.

## DISCUSSION

Using nationally representative data from the recently conducted NHFS in India, this study found that only 9 and 20 per 1,000 women aged 30–49 years in India undergo testing for BC and CC, respectively. Testing uptake rate was relatively higher among educated women, women from affluent households and those living in urban areas. We observed higher levels of socioeconomic and education-based inequality in the uptake of BC examination and CC testing. The magnitude of such inequality in the uptake of tests for early cancer detection varies across regions, states, and union territories. Higher levels of socioeconomic and education-based inequalities were observed in the Western and Northeastern regions. Higher education, higher SES, overweight, higher level of education in the community, and residence in the Southern region and urban areas were identified as potential determinants for the ever uptake of BC examination and CC testing. Cancer screening trials conducted in India also reported higher screening uptake among individuals with higher education and high socio-economic status.^29^^,^^30^ To our knowledge, this is the most comprehensive study that has investigated inequality in the uptake of BC examination and CC testing based on education and household SES at national and subnational levels in India.

Given that a significant percentage of cancer mortality among women in India is attributed to BC and CC in India,^4^^,^^7^^–^^11^ the finding that fewer than ten per 1,000 women aged 30–49 years underwent BC examination and 20 per 1,000 women received CC testing is concerning. Our study also revealed the poor uptake of tests even among women who are eligible and at high risk for these cancers. The low uptake of BC examination and CC testing is consistent with neighboring South Asian countries. In Bangladesh, the uptake of CC testing is about 6.1% among women aged 30–49 years^31^ and around 7% among women aged 30–49 years in Nepal.^32^^,^^33^ In 2016, India implemented an operational framework for mandatory BC and CC screening every 5 years for all eligible women (aged over 30 years) in 100 districts with a vision to expand this initiative to the entire country by 2017.^15^^,^^34^^,^^35^ However, only the Southern part of the country has the highest uptake rate of BC examination and CC testing than other parts of India as found in this study. Initiatives such as the Tamil Nadu Health System Project (TNHSP) might have helped increase the uptake of BC and CC screening in some regions.^36^

Consistent with findings from various studies,^17^^,^^19^^,^^21^^,^^22^^,^^37^ our study showed the uptake of BC examination and CC testing increases with improved SES. The extent of wealth-related inequality varied in the uptake of both BC examination and CC testing across different sub-national regions. Rural women exhibited higher levels of wealth-based inequality, indicating that the richest women in rural areas have relatively higher likelihood of undergoing tests for early cancer detection compared to the poorest women. This finding is in line with a previous study that reported that rural women with middle SES are less likely to undergo BC examination than those with high SES.^38^ Women from disadvantaged households are less likely to undergo tests for cancer detection mainly because of high diagnosis cost, as well as fear of cancer diagnosis and stigma related to cancer.^39^^,^^40^ The lack of comprehensive national health insurance scheme for prevention and screening often makes it difficult for disadvantaged women to undergo tests for cancer diagnosis. In 2018, the Government of India introduced a national public health insurance—known as Ayushman Bharat Pradhan Mantri Jan Arogya Yojana (PM-JAY)—which covers 500,000 Indian Rupees per family each year for secondary and tertiary level hospitalization and currently covers about 120 million poor and vulnerable families.^41^ Although many poor and vulnerable women are covered by health insurance, uptake of tests for early detection of BC and CC will not increase unless more efforts directed towards increasing awareness and health seeking behavior are initiated at community level. Therefore, health care schemes should include early diagnosis and screening within the basic packages of health insurance but intervention in terms of increasing awareness, local community engagement and increasing access should also be prioritized in order to expand the uptake of BC examination and CC testing.

This study observed significant education-based inequality in the uptake of BC examination and CC testing. Our research shows that women with higher education are more likely to undergo tests for cancer detection, which is in line with the findings of previous studies conducted in India.^29^^,^^42^^,^^43^ The extent of education-based inequality varied significantly across sub-dimensions, with a notably high inequality among rural women. This suggest that rural women with higher education levels are more likely to participate in cancer detection programs than uneducated counterparts. This finding aligns with a study conducted in Nepal in 2014, which found that literate women were more likely to participate in CC screening than their illiterate peers.^44^ In addition, educational status was found to be a significant factor for the uptake of tests for early cancer detection. These results highlight the need for improved access to education for women in order to make them more aware of their health needs. Furthermore, we identified community education level as one of the important contextual factors affecting the uptake of both BC examination and CC testing among Indian women, which is consistent with previous studies.^16^^,^^17^^,^^19^^,^^21^^,^^29^ Therefore, in order to minimize the burden of cancer and meet the target of cancer screening programs, the government of India must improve the women’s education rate as well as expand community-level health education activities.

Apart from household SES and education level, our study also identified other important demographic, health behavior and community-level risk factors, such as respondent’s age, marital status, higher BMI, parity, drinking, tobacco consumption, membership in the health insurance scheme, level of education in the community, type of place of residence, access to media, religion, and region of residence. In this study, we found that Hindu and Christian women were more likely to undergo BC examination and CC testing compared to Muslim women, consistent with other studies conducted in India in 2006 and 2015–16 respectively,^16^^,^^21^^,^^45^ with the exception of one study in India.^46^ Cultural or religious barriers may influence women to feel uncomfortable discussing their health issues with their doctor, which could decrease the uptake of early cancer detection, especially among Muslims.^47^ A previous review reported that Muslim women avoid CC screening because of shame,^48^ while others claimed that women avoid screening because they feel uncomfortable undressing in front of male doctors or nurses or discussing health issues with men.^39^^,^^49^ Ensuring a supportive and comfortable environment through female-centric services, improvements in professional training, and a focus on dignity and quality in care, may help to cater to the concerns of women who avoid screening due to shame and contribute to increasing overall uptake in cervical cancer screening programs. Place of residence is another important factor for the uptake of early cancer detection. Urban women have more opportunities to get tested for early cancer detection compared to rural women due to the proximity of clinics, logistical support (transportation, shorter waiting time, etc), SES, and other factors.^16^^,^^17^^,^^21^^,^^46^^,^^50^ Differences in the environment and services across states may explain variations in the uptake of BC examination and CC testing by state and place of residence. Furthermore, access to mass media, either TV or radio, has a significant link with early cancer detection. We found that those who have access to mass media had significantly higher odds of receiving early cancer diagnosis compared with women who do not have access to mass media.^46^ In addition, a previous study identified health system strengthening and political commitments as key factors for improving screening coverage.^51^ Community health workers with the proper training can play a crucial role in expanding self-breast examination and self-awareness among disadvantaged women. As women have taboos about discussing their health issues and undressing in front of male health professionals, the government should open health sections to female health professionals for gynecological issues.

This study’s key strength is its large sample size, which enables an in-depth examination of demographic subgroups. We can extrapolate these findings across all of India from the NFHS since it is a representative sample of the population. As there was a significant difference in the early detection of BC and CC uptake at different educational levels, it was therefore essential to study the magnitude of inequalities in BC examination and CC testing. To the best of our knowledge, this is the first study that discussed the extent and magnitude of education-based inequality in different subgroups. Despite several strengths, this study has a few limitations that warrant mentioning. First, the outcome measurement is subject to recall bias as well as social desirability bias. Second, the study was limited to women aged 30–49 years, so we could not obtain information on cancer testing for women aged over 50 years. Third, there was no information on the reason for cancer testing, which restricted us to distinguish between women who got screened as a preventive measure and who got tested as a diagnostic examination for suspected symptoms. Low screening program participation can be related to a number of issues, including a lack of information about the availability of screening services as well as a lack of convenient access to services. Along with these factors, people neglect to give enough attention to the screening program. Screening services should be easy to reach, and positive cases should be followed up regularly. Future studies should collect information on the reason for cancer screening. Finally, the cross-sectional design of this study precludes inference of a causal relationship between the covariates and outcome.

### Conclusion

The uptake of BC examination and CC testing is extremely low among Indian women aged 30–49 years. The uptake was relatively higher in the Southern region, while the Eastern region had the lowest uptake rate. Wide socioeconomic and education-based inequality in the uptake of tests for early cancer detection exists; the poor and those living in rural areas are less likely to undergo tests for early detection of BC and CC. However, the magnitude of such inequalities varies across urban and rural areas, regions, and states. These findings highlight an urgent need for multifaceted improvement, including but not limited to: basic education, cancer awareness, health insurance coverage and inclusion of essential cancer services in the benefit package, allowance for absence from work for cancer screening, transportation assistance, access to mass media, freedom to choose female healthcare providers, and introduction of new technology (eg, self-sampled human papillomavirus testing for CC screening). Without further efforts to improve uptake of, and reduce inequality in, BC and CC screening, Indian women will continue to face an unnecessarily high burden of these diseases.

## SUPPLEMENTARY MATERIAL

eTable 1. Details of covariates used in this study

eTable 2. Background characteristics of included Indian women by states and union territories

eTable 3. Number of women who underwent breast cancer examination and cervical cancer testing by household socioeconomic status

eTable 4. Number of women who underwent breast cancer examination and cervical cancer testing by their educational status

eTable 5. Uptake of breast cancer examination and cervical cancer test among Indian women from poorest and richest households

eTable 6. Uptake of breast cancer examination and cervical cancer test among Indian women by education status

eTable 7. Absolute and relative socioeconomic inequality in the uptake of breast cancer examination and cervical cancer test among Indian women across states and union territories

eTable 8. Absolute and relative education-based inequality in the uptake of breast cancer examination and cervical cancer test among Indian women across states and union territories

eTable 9. Socioeconomic and education-based relative inequalities in the uptake of breast cancer examination and cervical cancer test among Indian women

eFigure 1. Participant selection flowchart, the 2019–21 National Family Health Survey conducted in India

eFigure 2. Uptake of breast cancer examination and cervical cancer test among Indian women by socioeconomic status across states and union territories.

eFigure 3. Uptake of breast cancer examination and cervical cancer test among Indian women by educational status across states and union territories.

eFigure 4. Heatmap for the uptake of breast examination and cervical cancer test among Indian women (combination of socioeconomic status and education) at the national level.

## References

[1] Foreman KJ, Marquez N, Dolgert A, . Forecasting life expectancy, years of life lost, and all-cause and cause-specific mortality for 250 causes of death: reference and alternative scenarios for 2016–40 for 195 countries and territories. Lancet. 2018;392(10159):2052–2090. 10.1016/S0140-6736(18)31694-5 30340847 PMC6227505

[2] Bray F, Jemal A, Grey N, Ferlay J, Forman D. Global cancer transitions according to the Human Development Index (2008–2030): a population-based study. Lancet Oncol. 2012;13(8):790–801. 10.1016/S1470-2045(12)70211-5 22658655

[3] International Agency for Research on Cancer. World cancer report: cancer research for cancer prevention. Accessed March 1, 2023, https://publications.iarc.fr/Non-Series-Publications/World-Cancer-Reports/World-Cancer-Report-Cancer-Research-For-Cancer-Prevention-2020.

[4] Sung H, Ferlay J, Siegel RL, . Global cancer statistics 2020: GLOBOCAN estimates of incidence and mortality worldwide for 36 cancers in 185 countries. CA Cancer J Clin. 2021;71(3):209–249. 10.3322/caac.21660 33538338

[5] Mandal R, Basu P. Cancer screening and early diagnosis in low and middle income countries: current situation and future perspectives [Krebsvorsorge und Früherkennung in Ländern mit niedrigem und mittlerem Einkommen: Aktuelles Szenario und Zukunftsperspektiven]. Bundesgesundheitsblatt Gesundheitsforschung Gesundheitsschutz. Dec 2018;61(12):1505–1512. 10.1007/s00103-018-2833-9 30353287

[6] Lemp JM, De Neve JW, Bussmann H, . Lifetime prevalence of cervical cancer screening in 55 low- and middle-income countries. JAMA. 2020;324(15):1532–1542. 10.1001/jama.2020.16244 33079153 PMC7576410

[7] Ferlay J, Ervik M, Lam F, et al. Global cancer observatory: cancer today. Lyon: International Agency for Research on Cancer; 2020. International Agency for Research on Cancer. Available from: https://gco.iarc.fr/today, accessed [06 June 2023]. 2023.

[8] National Centre for Disease Informatics and Research. Report of National Cancer Registry Programme (2012–2016). 2020. Accessed August 12, 2023. https://www.ncdirindia.org/All_Reports/Report_2020/resources/NCRP_2020_2012_16.pdf.

[9] Budukh A, Thakur JS, Dikshit R, et al. Cancer Incidence and Mortality in Sangrur District, Punjab, India: 2017–2018 Tata Memorial Centre (TMC), Mumbai and Post Graduate Institute of Medical Education and Research (PGIMER), Chandigarh, India 2022. 2022. https://tmc.gov.in/tmh/pdf/Reports/Sangrur%20PBCR%20Report%202017-2018.pdf.

[10] Budukh A, Khanna D, Bagal S, et al. Cancer Incidence and Mortality in Varanasi District, Uttar Pradesh, India: 2018–2019 Tata Memorial Centre, Mumbai, India 2022. 2022. https://tmc.gov.in/tmh/pdf/Reports/Varanasi%20PBCR%20Detail%20report%20%202018-2019.pdf.

[11] Budukh A, Bagal S, Pandey N, et al. Cancer Incidence and Mortality in Muzaffarpur, Bihar State, India: 2018. Tata Memorial Centre (TMC), Mumbai, India 2023. 2023. https://tmc.gov.in/tmh/pdf/Reports/Muzaffarpur%20PBCR_2018.pdf.

[12] Pramesh CS, Badwe RA, Bhoo-Pathy N, . Priorities for cancer research in low- and middle-income countries: a global perspective. Nat Med. 2022;28(4):649–657. 10.1038/s41591-022-01738-x 35440716 PMC9108683

[13] Birnbaum JK, Duggan C, Anderson BO, Etzioni R. Early detection and treatment strategies for breast cancer in low-income and upper middle-income countries: a modelling study. Lancet Glob Health. 2018;6(8):e885–e893. 10.1016/S2214-109X(18)30257-2 30012269 PMC6214657

[14] Kakushadze Z, Raghubanshi R, Yu W. Estimating cost savings from early cancer diagnosis. Data. 2017;2(3):30. 10.3390/data2030030

[15] Ministry of Health and Family Welfare. Operational framework: Management of common cancers. 2016. Accessed June 12, 2023. https://main.mohfw.gov.in/sites/default/files/Operational%20Framework%20Management%20of%20Common%20Cancers_1.pdf.

[16] Krishnamoorthy Y, Ganesh K, Sakthivel M. Prevalence and determinants of breast and cervical cancer screening among women aged between 30 and 49 years in India: Secondary data analysis of National Family Health Survey–4. Indian J Cancer. 2022;59(1):54–64. 10.4103/ijc.IJC_576_19 33753601

[17] Singh S, Badaya S. Factors influencing uptake of cervical cancer screening among women in India: a hospital based pilot study. J Community Med Health Educ. 2012;2:157. 10.4172/2161-0711.1000157

[18] Muthuramalingam MR, Muraleedharan VR. Patterns in the prevalence and wealth-based inequality of cervical cancer screening in India. BMC Womens Health. 2023;23(1):337. 10.1186/s12905-023-02504-y 37365552 PMC10291770

[19] Negi J, Nambiar D. Intersectional social-economic inequalities in breast cancer screening in India: analysis of the National Family Health Survey. BMC Womens Health. 2021;21(1):324. 10.1186/s12905-021-01464-5 34493267 PMC8424809

[20] Nilima N, Mani K, Kaushik S, Rai SN. Cervical cancer screening and associated barriers among women in India: a generalized structural equation modeling approach. Cancers (Basel). 2022;14(13):3076. 10.3390/cancers14133076 35804848 PMC9264854

[21] Sen S, Khan PK, Wadasadawala T, Mohanty SK. Socio-economic and regional variation in breast and cervical cancer screening among Indian women of reproductive age: a study from National Family Health Survey, 2019–21. BMC Cancer. 2022;22(1):1279. 10.1186/s12885-022-10387-9 36476339 PMC9727878

[22] Srivastava S, Kurian K, Garg PR, . Prevalence and predictors of cervical cancer screening among reproductive age group women: evidence from cross-sectional study in Rohtak and Delhi. Asian Pac J Cancer Prev. 2022;23(8):2771–2777. 10.31557/APJCP.2022.23.8.2771 36037133 PMC9741889

[23] International Institute for Population Sciences, ICF. *National Family Health Survey (NFHS-5), 2019–21: India: Volume I*. 2021. National Family Health Survey, India.

[24] International Institute for Population Sciences and ICF. *National Family Health Survey (NFHS-5), 2019–21: India: Volume II*. 2021. Mumbai: International Institute for Population Sciences.

[25] TATA Memorial Center, National Cancer Grid of India. Consensus Evidence Based Resource Stratified Guidelines on Secondary Prevention of Cervical, Breast & Oral Cancers. 2019. Accessed April 19, 2023. https://tmc.gov.in/ncg/docs/PDF/DraftGuidelines/Preventive/3_%20NCG_INDIA_Rev_Preventive%20Oncology_Primary_Care.pdf.

[26] Choi B, Um TR, Lee KS. Factors related to cancer screening behaviors. Epidemiol Health. 2018;40:e2018011. 10.4178/epih.e2018011 29642655 PMC5968203

[27] Schlotheuber A, Hosseinpoor AR. Summary measures of health inequality: a review of existing measures and their application. Int J Environ Res Public Health. 2022;19(6):3697. 10.3390/ijerph19063697 35329383 PMC8992138

[28] Chen W, Qian L, Shi J, Franklin M. Comparing performance between log-binomial and robust Poisson regression models for estimating risk ratios under model misspecification. BMC Med Res Methodol. 2018;18(1):63. 10.1186/s12874-018-0519-5 29929477 PMC6013902

[29] Nene B, Jayant K, Arrossi S, . Determinants of women’s participation in cervical cancer screening trial, Maharashtra, India. Bull World Health Organ. 2007;85(4):264–272. 10.2471/BLT.06.031195 17546307 PMC2636321

[30] Budukh A, Maheshwari A, Bagal S, . Factors influencing women to participate in cervical cancer screening by providing menstrual pads: a population-based study from rural areas of Maharashtra state, India. Indian J Cancer. 2022;59(4):462–468. 10.4103/ijc.IJC_910_19 34380839

[31] World Health Organization, Directorate General of Health Services, National Institute of Preventive and Social Medicine. National STEPS survey for non-communicable diseases risk factors in Bangladesh 2018. 2018. Accessed April 29, 2023. https://cdn.who.int/media/docs/default-source/searo/bangladesh/publications/2018-national-steps-survey-for-non-communicable-diseases-risk-factors-in-bangladesh.pdf?sfvrsn=9155c4b5_3&download=true.

[32] Dhimal M, Bista B, Bhattarai S, et al. Report of Non communicable Disease Risk Factors: STEPS Survey Nepal 2019. 2020. Accessed July 10, 2023. https://www.who.int/docs/default-source/nepal-documents/ncds/ncd-steps-survey-2019-compressed.pdf.

[33] Rahman MS, Rahman MM, Acharya K, . Disparities and determinants of testing for early detection of cervical cancer among Nepalese women: evidence from a population-based survey. Cancer Epidemiol Biomarkers Prev. 2024;33(8):1046–1056. 10.1158/1055-9965.EPI-24-0037 38820125

[34] Kastor A, Mohanty SK. Disease-specific out-of-pocket and catastrophic health expenditure on hospitalization in India: do Indian households face distress health financing? PLoS One. 2018;13(5):e0196106. 10.1371/journal.pone.0196106 29746481 PMC5945043

[35] Directorate General of Health Services. National programme for prevention and control of cancer diabetes, cardiovascular diseases & stroke (NPCDCS), operational guidelines (revised: 2013-17). 2013. Accessed July 28, 2023. https://main.mohfw.gov.in/sites/default/files/Operational%20Guidelines%20of%20NPCDCS%20%28Revised%20-%202013-17%29_1.pdf.

[36] Tamilnadu Health System Project. Significant Interventions. Accessed July 11, 2023. https://tnhsp.org/tnhsp/significant-interventions.php.

[37] Patil P, Sarang B, Bhandarkar P, Ghoshal R, Roy N, Gadgil A. Does increase in women’s empowerment and socio-economic conditions affect uptake of breast cancer screening? Findings from NFHS (5), India. BMC Womens Health. 2023;23(1):7. 10.1186/s12905-022-02147-5 36611149 PMC9824936

[38] Akinyemiju TF. Socio-economic and health access determinants of breast and cervical cancer screening in low-income countries: analysis of the World Health Survey. PLoS One. 2012;7(11):e48834. 10.1371/journal.pone.0048834 23155413 PMC3498259

[39] Srinath A, van Merode F, Rao SV, Pavlova M. Barriers to cervical cancer and breast cancer screening uptake in low- and middle-income countries: a systematic review. Health Policy Plan. 2023;38(4):509–527. 10.1093/heapol/czac104 36525529 PMC10089064

[40] Nyblade L, Stockton M, Travasso S, Krishnan S. A qualitative exploration of cervical and breast cancer stigma in Karnataka, India. BMC Womens Health. Aug 2 2017;17(1):58. 10.1186/s12905-017-0407-x 28768506 PMC5541646

[41] Grewal H, Sharma P, Dhillon G, Munjal RS, Verma RK, Kashyap R. Universal health care system in India: an in-depth examination of the Ayushman Bharat Initiative. Cureus. 2023;15(6):e40733. 10.7759/cureus.40733 37485096 PMC10360977

[42] Mahalakshmi S, Suresh S. Barriers to cancer screening uptake in women: a qualitative study from Tamil Nadu, India. Asian Pac J Cancer Prev. 2020;21(4):1081–1087. 10.31557/APJCP.2020.21.4.1081 32334474 PMC7445965

[43] Dsouza JP, Van den Broucke S, Pattanshetty S, Dhoore W. Exploring the barriers to cervical cancer screening through the lens of implementers and beneficiaries of the national screening program: a multi-contextual study. Asian Pac J Cancer Prev. 2020;21(8):2209–2215. 10.31557/APJCP.2020.21.8.2209 32856846 PMC7771922

[44] Acharya Pandey R, Karmacharya E. Cervical cancer screening behavior and associated factors among women of Ugrachandi Nala, Kavre, Nepal. Eur J Med Res. 2017;22(1):32. 10.1186/s40001-017-0274-9 28927464 PMC5606016

[45] Grosse Frie K, Ramadas K, Anju GA, . Determinants of participation in a breast cancer screening trial in Trivandrum district, India. Asian Pac J Cancer Prev. 2013;14(12):7301–7307. 10.7314/APJCP.2013.14.12.7301 24460292

[46] Changkun Z, Bishwajit G, Ji L, Tang S. Sociodemographic correlates of cervix, breast and oral cancer screening among Indian women. PLoS One. 2022;17(5):e0265881. 10.1371/journal.pone.0265881 35544475 PMC9094566

[47] Thomas VN, Saleem T, Abraham R. Barriers to effective uptake of cancer screening among Black and minority ethnic groups. Int J Palliat Nurs. 2005;11(11):562–571. 10.12968/ijpn.2005.11.11.20096 16471043

[48] Petersen Z, Jaca A, Ginindza TG, . Barriers to uptake of cervical cancer screening services in low-and-middle-income countries: a systematic review. BMC Womens Health. 2022;22(1):486. 10.1186/s12905-022-02043-y 36461001 PMC9716693

[49] Shin HY, Song SY, Jun JK, Kim KY, Kang P. Barriers and strategies for cervical cancer screening: what do female university students know and want? PLoS One. 2021;16(10):e0257529. 10.1371/journal.pone.0257529 34610022 PMC8491915

[50] Tripathi N, Kadam YR, Dhobale RV, Gore AD. Barriers for early detection of cancer amongst Indian rural women. South Asian J Cancer. 2014;3(02):122–127. 10.4103/2278-330X.130449 24818108 PMC4014643

[51] Ong SK, Abe SK, Thilagaratnam S, . Towards elimination of cervical cancer–human papillomavirus (HPV) vaccination and cervical cancer screening in Asian National Cancer Centers Alliance (ANCCA) member countries. Lancet Reg Health West Pac. 2023;39:100860. 10.1016/j.lanwpc.2023.100860 37576906 PMC10415801

