# Supplementary material for: Regional Variations and Inequalities in Testing for Early Detection of Breast and Cervical Cancer: Evidence From a Nationally Representative Survey in India
Source: J Epidemiol. 2025 Mar 5;35(3):129–40. doi: 10.2188/jea.JE20240065 (PMC11821381; doi:10.2188/jea.JE20240065)
Supplement: Supplementary file 1 [file je-35-129-s001.pdf]

eTable 1. Details of covariates used in this study

Variable name	Detail	Categories
Individual-level		
Age group, years	Age of respondent	1= 30–34 years; 2= 35–39 years; 3= 40–44 years; 4= 45–49 years
Highest educational level	The highest educational level the respondent completed	1= No education (0 years of education); 2= Primary education; 3= Secondary education; 4= Higher education
Marital status	Current marital status of the respondent	1= Currently married; 2= Others (widowed/divorced/separated)
Body mass index	Body mass index (BMI) was estimated as weight (in kg) divided by the square value of height (in meters) of the respondents.	1= Underweight (BMI<18.5 kg/m^2); 2= Normal (BMI: 18.5 to <25 kg/m^2); 3= Overweight or Obese (BMI>25.0 kg/m^2)
Exposure to mass media	Exposure to mass media —a composite variable— was generated based on the respondent's self-reported information on the frequency of reading newspapers or magazines, listening radio, and watching television. The response options for these exposures to mass media were "not at all" (assigned the value 0), "less than once a week" (assigned the value 1), and "at least once a week" (assigned the value 2). Then the value of these questions was summed to get a composite value. The composite value ranged from 0 to 6 and categorized as "no access" for the value of 0, "minimum access" for the value of 1, "moderate access" for the value between 2-3, and "high access" for the value between 4-6.	1= No access; 2= Minimum access; 3= Moderate access; 4= Higher access
Age of respondent at first birth	How old was the respondent at her first childbirth?	1= 13–19 years; 2= 20–24 years; 3= 25 years and above
Number of children ever born	How many children do the respondents have?	1= None; 2= 1–2 children; 3= 3 or more children
Drinks alcohol	Alcohol use was defined as the current use of alcohol.	0= No; 1= Yes

Tobacco use	Tobacco use was defined as the current use of tobacco in any form (both smoking and smokeless tobacco products were included).	0= No; 1= Yes
Religion	In which religion the respondent belongs?	1= Hindu;<br>2= Muslim;<br>3= Christian;<br>4= Other (Sikh, Buddhist/neo-Buddhist, Jain, Jewish, Parsi/Zoroastrian, no religion, and other)
Health insurance	Are the respondents covered by any health scheme or any health insurance?	0= No; 1= Yes
Household-level		
Household socioeconomic status	Household socioeconomic status was assessed based on a proxy variable-wealth index. The wealth index is constructed using data on household assets and living conditions, typically through Principal Component Analysis. Households are then ranked by their wealth index scores and divided into five quintiles, with each quintile representing 20% of the population, from the poorest to the wealthiest. Details of the procedures for calculating the wealth index is presented elsewhere.[1]	1= Poorest; 2= Poorer;<br>3= Middle; 4= Richer;<br>5= Richest
Sex of household head	Sex of the main decision-making person of the respondent household.	1= Male; 2= Female
Community-level		
Place of residence	Place of residence of the respondent.	1= Urban areas; 2= Rural areas
Region	Which region do the respondents belong and the region categories as north region (states of Himachal Pradesh, Punjab, Uttarakhand, Haryana, Rajasthan, and four union territories Jammu and Kashmir, Chandigarh, National Capital Territory of Delhi, and Ladakh), south region (states of Andhra Pradesh, Karnataka, Kerala, Tamil Nadu, Telangana and as well as three union territories Lakshadweep, Puducherry, Andaman and Nicobar Islands), east region (the states of Bihar, West Bengal, Jharkhand, and Odisha), western region (States of Gujarat, Maharashtra, Goa and one union territory Dadra & Nagar Haveli and Daman & Diu), central region (states of Chhattisgarh, Uttar Pradesh, and Madhya Pradesh), northeast region (States of Sikkim, Arunachal Pradesh, Nagaland, Manipur, Mizoram, Tripura, Meghalaya, and Assam).	1= North;<br>2= Central;<br>3= East;<br>4= Northeast;<br>5= West;<br>6= South
Community Education	Community-level education was calculated based on women's education level in the community and the national aggregate. The number of years of formal education for each woman was derived from the	1= Low;

	survey data. The national aggregate was determined by calculating the median years of formal education for all women included in the sample. According to the NFHS-5, the median duration of female schooling was reported to be 5 years. Clusters (communities) were categorized as having either lower or higher levels of female education based on whether their median level of female education was lower or higher than the national average, respectively.	2= High

BMI, body mass index.
Data Source: National Family Health Survey (NFHS-5), 2019-21.
[1] Rutstein SO, Johnson K. The DHS wealth index. DHS comparative reports no. 6. Calverton, Maryland: ORC Macro, 2004

eTable 2. Background characteristics of included Indian women by states and union territories

Characteristics	Number of participants (%)	Uptake per 1,000 women (95% CI)	
		Breast cancer examination	Cervical cancer test
**State**			
Himachal Pradesh	6,067 (0.6)	4.3 (2.3–8.1)	8.9 (5.7–13.8)
Punjab	11,265 (2.3)	3.2 (2.2–4.8)	26.1 (22.9–29.9)
Uttarakhand	6,427 (0.8)	1.8 (0.7–4.3)	4.4 (2.5–7.7)
Haryana	10,585 (1.9)	3.0 (2.0–4.7)	8.2 (6.3–10.6)
Rajasthan	19,291 (5.7)	1.7 (1.2–2.4)	4.2 (3.4–5.2)
Uttar Pradesh	39,324 (14.5)	3.7 (3.2–4.3)	16.0. (14.9–17.1)
Bihar	17,894 (7.4)	3.2 (2.6–4.0)	8.3 (7.3–9.5)
Sikkim	1,797 (0.1)	1.2 (0.0–76.6)	6.2 (0.9–39.0)
Arunachal Pradesh	10,243 (0.1)	3.4 (0.5–22.9)	8.5 (2.5–28.3)
Nagaland	4,991 (0.1)	2.8 (0.4–18.1)	2.8 (0.4–18.1)
Manipur	4,382 (0.2)	15.7 (8.7–28.2)	21.6 (13.1–35.5)
Mizoram	3,996 (0.1)	26.4 (13.4–51.3)	70.4 (46.8–104.6)
Tripura	3,894 (0.3)	3.6 (1.4–9.2)	6.7 (3.3–13.3)
Meghalaya	5,902 (0.2)	5.1 (2.0–13.3)	6.4 (2.7–15.1)
Assam	17,431 (2.7)	1.8 (1.1–2.9)	2.0 (1.3–3.2)
West Bengal	10,796 (8.7)	1.6 (1.2–2.1)	2.0 (1.6–2.6)
Jharkhand	12,104 (2.4)	1.1 (0.6–2.1)	4.8 (3.5–6.5)
Odisha	14,344 (3.5)	2.1 (1.5–3.1)	9.3 (7.8–11.2)
Chhattisgarh	13,473 (2.3)	2.0 (1.3–3.3)	2.8 (1.9–4.3)
Madhya Pradesh	22,111 (5.4)	5.5 (4.6–6.7)	8.7 (7.4–10.1)
Gujarat	17,279 (4.9)	1.3 (0.8–1.9)	2.4 (1.7–3.2)
Maharashtra	17,749 (9.7)	13.9 (12.7–15.2)	24.8 (23.2–26.5)
Andhra Pradesh	6,096 (4.5)	8.0 (6.7–9.5)	47.9 (44.7–51.3)
Karnataka	16,068 (5.7)	3.3 (2.6–4.3)	5.5 (4.6–6.6)
Goa	1,152 (0.2)	13.0 (6.5–25.8)	12.1 (5.9–24.7)
Kerala	6,584 (3.1)	24.5 (21.7–27.6)	35.6 (32.3–39.3)
Tamil Nadu	14,552 (7.0)	58.3 (55.4–61.3)	101.3 (97.5–105.1)
Telangana	14,721 (2.9)	3.6 (2.6–4.9)	35.0 (31.6–38.8)
Union territories			
Jammu & Kashmir	10,748 (0.9)	2.8 (1.5–5.5)	4.8 (2.9–7.9)
Chandigarh	368 (0.1)	0.0 (0.0–0.0)	16.0 (6.5–38.8)
NCT of Delhi[a]	5,214 (1.5)	3.2 (2.0–5.2)	7.4 (5.4–10.2)
Dadra[b]	1,318 (0.0)	2.4 (0.1–76.7)	4.2 (0.3–57.5)
Lakshadweep	679 (0.0)	5.2 (0.0–571.0)	16.9 (0.8–273.1)
Puducherry	2,111 (0.1)	41.6 (25.7–66.6)	74.6 (52.4–105.2)
Andaman[c]	1,399 (0.0)	15.8 (3.7–65.8)	24.4 (7.5–76.2)
Ladakh	1,163 (0.0)	1.7 (0.0–446.5)	2.8 (0.0–253.3)

CI, confidence interval.
[a]National Capital Territory of Delhi
[b]Dadra & Nagar Haveli and Daman & Diu
[c]Andaman & Nicobar Islands.

eTable 3. Number of women who underwent breast cancer examination and cervical cancer testing by household socioeconomic status

Characteristics	Breast cancer examination					Cervical cancer testing				
	Poorest quintile	Poorer quintile	Middle quintile	Richer quintile	Richest quintile	Poorest quintile	Poorer quintile	Middle quintile	Richer quintile	Richest quintile
**States**										
Himachal Pradesh	-	1	6	6	7	1	7	10	7	19
Punjab	-	1	3	-	27	1	9	25	57	198
Uttarakhand	-	2	2	6	2	1	1	2	9	8
Haryana	1	2	6	6	20	2	5	12	18	55
Rajasthan	2	3	8	5	9	6	10	18	14	21
Uttar pradesh	36	29	31	21	30	194	170	118	80	89
Bihar	20	15	6	2	3	47	38	15	10	9
Sikkim	-	-	-	1	1	-	2	1	4	2
Arunachal pradesh	4	7	13	11	3	13	21	27	22	9
Nagaland	2	5	5	3	1	5	6	9	-	1
Manipur	6	11	16	13	3	8	32	18	20	5
Mizoram	3	6	17	23	22	4	10	47	80	77
Tripura	4	1	3	1	-	7	6	7	3	-
Meghalaya	3	2	1	4	2	7	5	5	6	1
Assam	5	8	5	8	4	12	4	8	8	3
West bengal	-	10	7	3	1	3	14	7	1	2
Jharkhand	4	5	2	2	1	13	13	14	12	5
Odisha	9	8	5	-	5	38	28	30	13	12
Chhattisgarh	10	5	2	5	5	14	7	7	6	5
Madhya pradesh	19	20	14	18	19	41	38	32	22	24
Gujarat	4	3	5	6	9	4	4	12	9	17
Maharashtra	12	29	33	29	57	30	67	85	74	87
Andhra pradesh	2	9	11	16	8	10	41	82	123	46
Karnataka	1	12	13	14	7	7	17	29	22	12
Goa	-	-	3	3	8	-	-	2	4	8
Kerala	1	1	23	57	69	1	7	39	90	103
Tamil nadu	48	136	240	218	191	72	260	445	432	294
Telangana	2	13	16	19	12	19	106	204	149	64
**Union territories**										
Jammu & kashmir	4	7	9	6	5	8	8	12	16	12
Chandigarh	-	-	-	-	-	-	-	-	-	6
NCT of delhi[a]	-	1	-	1	18	-	1	-	7	34
Dadra[b]	-	-	-	1	2	-	-	1	2	3
Lakshadweep	-	-	-	4	-	-	1	2	6	3
Puducherry	-	1	7	10	29	2	6	16	14	51
Andaman[c]	12	12	25	10	3	15	14	28	8	7
Ladakh	1	-	1	-	-	1	1	-	1	-

[a]National Capital Territory of Delhi

[b]Dadra & Nagar Haveli and Daman & Diu

[c]Andaman & Nicobar Islands.

'-' indicates that the number of women who received cancer screening is zero in those subgroups.

Household socioeconomic status was measured using the wealth scores. The households were ranked based on these scores and subsequently divided into quintiles, each representing 20% of the population. These quintiles were categorized as follows: poorest (lowest 20%), poorer, middle, richer, and richest (highest 20%). Inequality assessment was not performed in a state or union territories if the number of women who received cancer screening is zero for any of the subgroups.

eTable 4. Number of women who underwent breast cancer examination and cervical cancer testing by their educational status

Characteristics	Breast cancer examination				Cervical cancer testing			
	No education	Primary education	Secondary education	Higher education	No education	Primary education	Secondary education	Higher education
**State**								
Himachal Pradesh	-	1	15	4	2	6	28	8
Punjab	1	3	21	6	53	37	148	52
Uttarakhand	5	1	4	2	5	6	9	1
Haryana	8	6	15	6	18	16	48	10
Rajasthan	13	4	8	2	36	9	20	4
Uttar Pradesh	60	22	41	24	297	90	195	69
Bihar	29	3	11	3	69	14	32	4
Sikkim	-	-	2	-	2	1	5	1
Arunachal Pradesh	8	4	18	8	16	18	47	11
Nagaland	1	1	11	3	5	4	11	1
Manipur	3	4	23	19	8	13	44	18
Mizoram	2	7	49	13	6	22	157	33
Tripura	-	3	6	-	3	9	10	1
Meghalaya	2	1	4	5	7	6	8	3
Assam	5	4	17	4	5	9	19	2
West Bengal	5	2	12	2	10	4	12	1
Jharkhand	6	4	4	-	20	9	26	2
Odisha	5	7	13	2	31	27	57	6
Chhattisgarh	11	5	7	4	18	5	14	2
Madhya Pradesh	37	13	24	16	72	17	51	17
Gujarat	12	-	12	3	16	4	19	7
Maharashtra	16	17	98	29	60	49	195	39
Andhra Pradesh	17	6	20	3	141	49	102	10
Karnataka	14	8	21	4	34	14	32	7
Goa	-	1	10	3	1	2	8	3
Kerala	-	3	104	44	-	15	169	56
Tamil Nadu	108	162	444	119	192	304	796	211
Telangana	33	4	23	2	360	59	105	18
**Union territories**								
Jammu & Kashmir	18	-	11	2	24	2	26	4
Chandigarh	-	-	-	-	2	-	3	1
NCT of delhi[a]	2	1	10	7	7	2	16	17
Dadra[b]	-	-	2	1	1	-	4	1
Lakshadweep	-	-	4	-	1	1	9	1
Puducherry	2	4	20	21	6	6	40	37
Andaman[c]	9	3	46	4	10	6	50	6
Ladakh	2	-	-	-	1	-	2	-

[a]National Capital Territory of Delhi

[b]Dadra & Nagar haveli and daman

[c]Andaman & Nicobar Islands.

'-' indicates that the number of women who received cancer screening is zero in those subgroups.

Respondents' education level was classified as no education (0 years of schooling), primary education (1–5 years of schooling), secondary education (6–12 years of schooling), and higher education (13 or more years of schooling). Inequality assessment was not performed in a state or union territories if the number of women who received cancer screening is zero for any of the subgroups.

eTable 5. Uptake of breast cancer examination and cervical cancer test among Indian women from poorest and richest households

Characteristics	Uptake per 1,000 women (95% CI)			
	Breast cancer examination		Cervical cancer test	
	Poorest quintile	Richest quintile	Poorest quintile	Richest quintile
**National**				
**Place of residence**				
Urban areas	4 (3–4)	13 (13–14)	10 (9–11)	25 (24–26)
Rural areas				
**Region**	11 (8–15)	15 (14–16)	12 (9–17)	25 (24–27)
North	3 (3–4)	10 (9–11)	10 (9–11)	24 (22–26)
Central				
East	1 (0–3)	4 (3–5)	3 (2–6)	14 (12–15)
Northeast	3 (3–4)	5 (4–6)	13 (12–15)	12 (10–14)
West	2 (1–2)	3 (2–5)	5 (4–6)	7 (5–9)
South	1 (1–3)	13 (7–24)	2 (1–4)	22 (13–35)
**State**				
Himachal Pradesh	–	–	0 (0–999)	17 (10–30)
Punjab	–	–	16 (2–101)	29 (25–34)
Uttarakhand	–	–	6 (1–44)	8 (4–16)
Haryana	7 (1–67)	3 (2–6)	11 (2–66)	9 (6–13)
Rajasthan	1 (0–3)	4 (2–6)	2 (1–5)	7 (5–10)
Uttar Pradesh	4 (3–5)	4 (3–5)	19 (17–21)	13 (11–16)
Bihar	3 (2–5)	5 (3–10)	8 (6–10)	12 (8–19)
Arunachal Pradesh	2 (0–280)	4 (0–924)	6 (0–114)	15 (0–456)
Nagaland	1 (0–470)	2 (0–803)	–	–
Manipur	10 (1–61)	14 (2–115)	10 (2–61)	21 (3–116)
Mizoram	8 (0–606)	49 (18–127)	10 (0–538)	149 (85–247)
Meghalaya	2 (0–33)	24 (3–154)	4 (0–30)	10 (1–177)
Assam	1 (0–3)	8 (3–22)	2 (1–4)	5 (1–19)
West Bengal	–	–	1 (0–1)	2 (1–5)
Jharkhand	1 (0–2)	2 (0–9)	2 (1–5)	6 (2–14)
Odisha	–	–	9 (7–13)	11 (6–19)
Chhattisgarh	2 (1–5)	4 (2–11)	3 (1–6)	4 (1–10)
Madhya Pradesh	3 (2–5)	9 (6–13)	6 (4–8)	11 (8–16)
Gujarat	1 (0–5)	2 (1–3)	1 (0–5)	3 (2–5)
Maharashtra	9 (6–13)	26 (23–30)	19 (14–25)	37 (33–41)
Andhra Pradesh	9 (4–21)	11 (7–16)	36 (24–53)	52 (44–61)
Karnataka	1 (0–5)	3 (1–5)	6 (3–11)	4 (2–7)
Kerala	24 (5–103)	30 (25–35)	24 (5–103)	40 (35–46)
Tamil Nadu	75 (60–92)	67 (61–74)	101 (84–121)	102 (94–110)
Telangana	2 (0–16)	4 (2–8)	23 (12–42)	29 (23–37)
**Union territories**				
Jammu & Kashmir	4 (1–24)	2 (0–9)	5 (1–26)	4 (2–12)
Puducherry	–	–	181 (28–625)	68 (39–117)
Andaman[a]	36 (1–630)	10 (0–431)	53 (2–573)	35 (4–268)

CI, confidence interval.

[a]Andaman & Nicobar Islands.

'-' indicates that the number of women who received cancer screening is zero in those subgroups.

Household socioeconomic status was measured using the wealth scores. The households were ranked based on these scores and subsequently divided into quintiles, each representing 20% of the population. These quintiles were categorized as follows: poorest (lowest 20%), poorer, middle, richer, and richest (highest 20%).

eTable 6. Uptake of breast cancer examination and cervical cancer test among Indian women by education status

Characteristics	Uptake per 1,000 women (95% CI)			
	Breast cancer examination		Cervical cancer test	
	No education	Higher education	No education	Higher education
**National**	4 (4–5)	16 (15–17)	14 (14–15)	25 (23–27)
**Place of residence**				
Urban areas	6 (5–7)	17 (16–19)	16 (14–18)	26 (24–28)
Rural areas	4 (4–4)	13 (11–15)	14 (13–15)	23 (20–26)
**Region**				
North	1 (1–2)	3 (2–5)	6 (5–7)	12 (10–15)
Central	3 (3–4)	8 (7–11)	12 (11–13)	17 (15–21)
East	2 (2–3)	3 (2–5)	6 (5–7)	4 (3–6)
Northeast	1 (0–3)	13 (7–23)	2 (1–4)	12 (6–21)
West	5 (4–6)	19 (16–23)	10 (9–12)	22 (19–26)
South	12 (11–14)	33 (29–36)	42 (39–45)	50 (46–54)
**State**				
Himachal Pradesh	–	–	2 (0–29)	13 (6–32)
Punjab	0 (0–4)	4 (2–10)	19 (14–27)	34 (25–47)
Uttarakhand	3 (1–10)	2 (0–14)	2 (1–10)	3 (1–15)
Haryana	2 (1–6)	4 (1–10)	6 (3–11)	6 (3–14)
Rajasthan	1 (1–2)	2 (0–6)	4 (3–5)	5 (2–10)
Uttar Pradesh	3 (2–4)	7 (5–9)	14 (13–16)	19 (16–23)
Bihar	3 (3–4)	6 (3–12)	8 (7–10)	7 (4–14)
Sikkim	–	–	2 (0–978)	23 (1–269)
Arunachal Pradesh	3 (0–88)	10 (0–390)	4 (0–71)	13 (0–341)
Nagaland	1 (0–794)	6 (0–171)	5 (0–176)	1 (0–972)
Manipur	8 (1–82)	30 (11–80)	23 (6–90)	29 (10–78)
Mizoram	4 (0–754)	60 (17–192)	15 (0–343)	95 (35–233)
Tripura	–	–	4 (0–34)	5 (0–156)
Meghalaya	1 (0–81)	34 (9–116)	4 (0–43)	14 (2–98)
Assam	1 (0–3)	6 (2–17)	1 (0–3)	3 (1–13)
West Bengal	1 (1–2)	2 (1–5)	3 (2–4)	1 (0–4)
Jharkhand	–	–	3 (2–5)	2 (0–13)
Odisha	1 (1–3)	2 (0–9)	8 (5–11)	8 (4–17)
Chhattisgarh	2 (1–4)	6 (2–17)	3 (2–6)	2 (0–12)
Madhya Pradesh	4 (3–6)	15 (10–23)	7 (6–10)	17 (12–26)
Gujarat	–	–	2 (1–4)	5 (3–10)
Maharashtra	8 (6–10)	26 (22–31)	17 (14–20)	29 (25–34)
Andhra Pradesh	6 (5–8)	8 (4–16)	50 (45–55)	20 (13–30)
Karnataka	3 (2–5)	6 (3–11)	7 (5–9)	6 (3–11)
Goa	–	–	7 (0–163)	8 (1–48)
Tamil Nadu	51 (45–59)	60 (53–68)	85 (76–94)	96 (87–106)
Telangana	4 (2–6)	2 (0–9)	43 (38–49)	16 (9–27)
**Union territories**				
Jammu & Kashmir	–	–	6 (3–12)	2 (0–20)
NCT of Delhi[a]	2 (0–7)	4 (2–10)	8 (4–15)	12 (7–20)
Lakshadweep	–	–	54 (0–1000)	11 (0–997)
Puducherry	17 (2–160)	45 (18–107)	61 (17–191)	86 (45–158)
Andaman[b]	17 (0–461)	7 (0–843)	29 (1–378)	30 (1–430)

CI, confidence interval.

[a]National Capital Territory of Delhi.

[b]Andaman & Nicobar Islands.

'-' indicates that the number of women who received cancer screening is zero in those subgroups.

Respondents' education level was classified as no education (0 years of schooling), primary education (1–5 years of schooling), secondary education (6–12 years of schooling), and higher education (13 or more years of schooling).

eTable 7. Absolute and relative socioeconomic inequality in the uptake of breast cancer examination and cervical cancer test among Indian women across states and union territories

Characteristics	Breast cancer examination		Cervical cancer test	
	SII (95% CI)	RCI (95% CI)	SII (95% CI)	RCI (95% CI)
**State**				
Himachal Pradesh	-	-	1.4 (0.2–2.5)*	12.9 (-7.5 to 33.3)
Punjab	-	-	1.5 (0.3–2.7)*	11.0 (1.3–20.8)*
Uttarakhand	-	-	1.1 (-0.1 to 2.2)	42.5 (22.0–63.0)†
Haryana	0.0 (-0.4 to 0.5)	2.5 (-20.4 to 25.4)	0.6 (-0.1 to 1.3)	11.7 (-1.2 to 24.5)
Rajasthan	0.3 (0.0 to 0.7)	17.5 (-3.8 to 38.8)	0.6 (0.1–1.0)*	9.1 (-6.4 to 24.5)
Uttar pradesh	0.0 (-0.2 to 0.2)	5.7 (-4.8 to 16.3)	-0.7 (-1.2 to -0.2)§	-4.3 (-9.7 to 1.2)
Bihar	-0.1 (-0.5 to 0.3)	-6.5 (-23.4 to 10.5)	0.2 (-0.4 to 0.8)	0.6 (-9.1 to 10.2)
Arunachal pradesh	0.5 (0.0–1.0)*	25.7 (8.5–42.9)§	0.8 (0.0–1.5)	17.0 (4.3–29.7)§
Nagaland	0.3 (-0.2 to 0.8)	25.2 (-1.0 to 51.3)	-	-
Manipur	2.0 (0.2–3.7)*	32.2 (17.4–47.1)†	1.1 (-0.7 to 2.9)	18.7 (6.4–31.1)§
Mizoram	4.9 (1.9–7.9)§	20.5 (6.0–34.9)§	17.2 (12.3–22.1)†	32.4 (24.5–40.4)†
Meghalaya	1.3 (-0.1 to 2.6)	23.3 (-11.9 to 58.4)	0.6 (-0.6 to 1.8)	15.5 (-9.9 to 41.0)
Assam	0.3 (0.1–0.6)*	30.8 (9.4–52.2)§	0.2 (-0.1 to 0.5)	13.8 (-9.1 to 36.6)
West bengal	-	-	0.0 (-0.2 to 0.2)	1.1 (-15.1 to 17.4)
Jharkhand	0.0 (-0.2 to 0.3)	12.9 (-16.1 to 42.0)	0.7 (0.3–1.2)§	27.5 (14.4–40.6)†
Odisha	-	-	0.1 (-0.5 to 0.7)	5.6 (-4.5 to 15.7)
Chhattisgarh	0.2 (-0.1 to 0.6)	6.8 (-14.9 to 28.5)	0.1 (-0.3 to 0.6)	1.6 (-19.4 to 22.6)
Madhya pradesh	0.7 (0.3–1.2)§	12.6 (-1.6 to 26.9)	0.6 (0.1–1.2)*	3.7 (-7.3 to 14.8)
Gujarat	0.2 (-0.1 to 0.4)	16.3 (-6.7 to 39.2)	0.3 (0.0–0.6)*	20.9 (5.9–36.0)§
Maharashtra	2.1 (0.9–3.4)§	21.8 (10.0–33.7)†	2.1 (0.7–3.6)§	11.4 (3.5–19.3)§
Andhra pradesh	0.4 (-0.5 to 1.3)	2.8 (-15.3 to 20.8)	3.5 (1.6–5.4)†	12.8 (5.3–20.3)§
Karnataka	-0.2 (-0.6 to 0.3)	6.0 (-11.0 to 22.9)	-0.2 (-0.6 to 0.3)	4.7 (-12.3 to 21.8)
Kerala	1.7 (0.2–3.2)*	16.6 (7.4–25.8)†	1.5 (-0.3 to 3.2)	12.4 (4.9–19.9)§
Tamil nadu	0.8 (-0.9 to 2.5)	-0.1 (-5.4 to 5.2)	-0.1 (-2.2 to 2.0)	-1.0 (-5.2 to 3.3)
Telangana	0.0 (-0.3 to 0.4)	6.5 (-8.3 to 21.2)	-0.7 (-1.9 to 0.5)	-2.5 (-7.5 to 2.5)
**Union territories**				
Jammu & kashmir	-0.2 (-0.5 to 0.2)	-10.5 (-27.8 to 6.9)	-0.1 (-0.5 to 0.4)	-1.5 (-16.4 to 13.3)
Puducherry	-	-	-4.6 (-11.8 to 2.5)	3.5 (-16.6 to 23.6)
Andaman[a]	-1.4 (-3.7 to 0.9)	-32.1 (-44.9 to -19.3)†	-1.7 (-5.1 to 1.6)	-29.7 (-42.4 to -17.0)†

CI, confidence interval; RCI, relative concentration index; SII, Slope index of inequality.

*P<0.05; §P<0.01; †P<0.001.

[a]Andaman & Nicobar Islands.

'-' indicates that the number of women who received cancer screening is zero in those subgroups.

Household socioeconomic status was measured using the wealth scores. The households were ranked based on these scores and subsequently divided into quintiles, each representing 20% of the population. These quintiles were categorized as follows: poorest (lowest 20%), poorer, middle, richer, and richest (highest 20%).

eTable 8. Absolute and relative education-based inequality in the uptake of breast cancer examination and cervical cancer test among Indian women across states and union territories

Characteristics	Breast cancer examination		Cervical cancer test	
	SII (95% CI)	RCI (95% CI)	SII (95% CI)	RCI (95% CI)
**State**				
Himachal Pradesh	-	-	1.5 (0.4–2.7)§	21.4 (2.3–40.4)*
Punjab	0.6 (0.3–1.0)§	29.3 (15.4–43.3)†	1.7 (0.5–3.0)§	9.9 (1.7–18.1)*
Uttarakhand	-0.1 (-0.6 to 0.3)	1.4 (-29.8 to 32.7)	0.2 (-0.5 to 1.0)	2.0 (-19.9 to 23.9)
Haryana	0.1 (-0.3 to 0.5)	-5.2 (-25.4 to 15.0)	0.2 (-0.4 to 0.8)	-3.4 (-15.3 to 8.5)
Rajasthan	0.2 (-0.1 to 0.6)	-2.7 (-27.5 to 22.1)	0.3 (-0.2 to 0.7)	-9.9 (-25.4 to 5.6)
Uttar Pradesh	0.3 (0.1–0.6)*	16.4 (6.7–26.0)§	0.7 (0.2–1.3)§	11.6 (6.8–16.5)†
Bihar	0.0 (-0.4 to 0.5)	-2.9 (-21.2 to 15.4)	0.0 (-0.7 to 0.8)	-6.2 (-18.9 to 6.6)
Sikkim	-	-	2.5 (-2.4 to 7.4)	-4.7 (-46.1 to 36.7)
Arunachal Pradesh	0.4 (-0.2 to 0.9)	19.0 (-1.3 to 39.3)	1.1 (0.4–1.8)§	13.4 (2.7–24.1)*
Nagaland	0.6 (-0.1 to 1.2)	36.2 (13.3–59.0)§	-0.5 (-1.1 to 0.1)	3.6 (-27.1 to 34.2)
Manipur	2.7 (0.6–4.7)*	36.1 (21.3–51.0)†	0.5 (-1.8 to 2.8)	12.6 (0.0–25.2)
Mizoram	6.2 (2.6–9.8)§	10.3 (-3.7 to 24.3)	8.0 (3.5–12.5)†	8.9 (1.6–16.2)*
Tripura	-	-	-0.2 (-1.2 to 0.8)	0.5 (-23.2 to 24.3)
Meghalaya	1.9 (0.0–3.7)*	35.2 (-5.6 to 76.1)	0.7 (-0.6 to 1.9)	-6.6 (-34.7 to 21.6)
Assam	0.3 (0.0–0.6)*	22.0 (1.9–42.2)*	0.2 (-0.1 to 0.4)	12.8 (-5.4 to 31.1)
West Bengal	0.2 (-0.1 to 0.4)	22.8 (2.7–43.0)*	-0.1 (-0.4 to 0.2)	-3.4 (-26.4 to 19.6)
Jharkhand	-	-	0.5 (0.0–0.9)*	12.6 (-0.1 to 25.3)
Odisha	0.2 (-0.1 to 0.5)	11.2 (-9.2 to 31.6)	0.3 (-0.3 to 1.0)	6.0 (-2.6 to 14.6)
Chhattisgarh	0.2 (-0.2 to 0.5)	5.5 (-19.0 to 29.9)	-0.1 (-0.5 to 0.3)	0.8 (-19.0 to 20.5)
Madhya Pradesh	0.6 (0.1–1.1)*	6.3 (-9.5 to 22.2)	0.8 (0.1–1.4)*	3.9 (-7.7 to 15.4)
Gujarat	-	-	0.2 (-0.2 to 0.6)	7.5 (-10.2 to 25.1)
Maharashtra	2.0 (0.7–3.3)§	38.1 (27.6–48.6)†	1.2 (-0.2 to 2.6)	18.1 (9.6–26.6)†
Andhra Pradesh	0.6 (-0.3 to 1.5)	6.6 (-11.7 to 24.9)	-0.8 (-2.8 to 1.1)	-1.2 (-8.4 to 6.0)
Karnataka	0.1 (-0.4 to 0.6)	4.7 (-12.3 to 21.7)	-0.3 (-0.9 to 0.2)	-1.7 (-14.5 to 11.2)
Goa	-	-	-1.2 (-3.8 to 1.5)	-13.6 (-41.7 to 14.6)
Tamil Nadu	0.5 (-1.3 to 2.3)	-0.6 (-5.1 to 3.9)	0.7 (-1.4 to 2.8)	1.6 (-2.1 to 5.4)
Telangana	0.0 (-0.4 to 0.3)	5.4 (-7.7 to 18.5)	-3.7 (-5.1 to -2.3)†	-13.3 (-18.6 to -8.1)†
**Union territories**				
Jammu & Kashmir	-	-	-0.3 (-0.8 to 0.2)	-5.7 (-19.7 to 8.4)
NCT of delhi[a]	0.4 (-0.1 to 0.9)	6.5 (-16.2 to 29.3)	0.7 (-0.3 to 1.7)	11.9 (-5.9 to 29.6)
Lakshadweep	-	-	-1.0 (-5.1 to 3.0)	-11.7 (-43.2 to 19.8)
Puducherry	2.0 (-2.8 to 6.9)	22.8 (4.0–41.5)*	3.4 (-3.2 to 10.0)	18.7 (5.5–32.0)§
Andaman[b]	-0.2 (-1.7 to 1.2)	-30.0 (-38.2 to -21.8)†	0.5 (-3.6 to 4.6)	-26.8 (-35.8 to -17.8)†

CI, confidence interval; RCI, relative concentration index; SII, Slope index of inequality.

**P*<0.05; §*P*<0.01; †*P*<0.001.

[a]National Capital Territory of Delhi

[b] Andaman & Nicobar Islands.

'-' indicates that the number of women who received cancer screening is zero in those subgroups.

Respondents' education level was classified as no education (0 years of schooling), primary education (1–5 years of schooling), secondary education (6–12 years of schooling), and higher education (13 or more years of schooling).

eTable 9. Socioeconomic and education-based relative inequalities in the uptake of breast cancer examination and cervical cancer test among Indian women

Characteristics	Relative index of inequality (95% confidence interval)	
	Breast cancer examination	Cervical cancer test
Socioeconomic inequality[a]		
National	3.72 (3.27–4.24)†	2.52 (2.32–2.74)†
Place of residence		
Urban areas	1.61 (1.35–1.93)†	1.23 (1.08–1.40)§
Rural areas	3.39 (2.83–4.07)†	3.15 (2.82–3.52)†
Region		
North	4.88 (2.14–11.15)†	7.50 (4.74–11.85)†
Central	1.77 (1.21–2.59)§	0.84 (0.67–1.04)
East	1.27 (0.72–2.25)	1.12 (0.78–1.60)
Northeast[b]	-	-
West	4.90 (3.35–7.18)†	2.31 (1.77–3.01)†
South	1.63 (1.34–1.98)†	1.23 (1.08–1.40)§
Education-based inequality[c]		
National	4.84 (4.23–5.55)†	2.12 (1.95–2.31)†
Place of residence		
Urban areas	3.35 (2.70–4.15)†	1.65 (1.43–1.91)†
Rural areas	4.18 (3.46–5.05)†	2.09 (1.86–2.35)†
Region		
North	3.59 (1.86–6.95)†	2.94 (2.09–4.14)†
Central	2.59 (1.76–3.83)†	1.58 (1.27–1.97)†
East	1.31 (0.74–2.32)	0.83 (0.58–1.20)
Northeast[b]	-	-
West	6.30 (4.25–9.34)†	2.47 (1.89–3.24)†
South	2.87 (2.40–3.42)†	1.22 (1.09–1.37)§

*$P<0.05$; §$P<0.01$; †$P<0.001$.

[a]Household socioeconomic status was measured using the wealth scores. The households were ranked based on these scores and subsequently divided into quintiles, each representing 20% of the population. These quintiles were categorized as follows: poorest (lowest 20%), poorer, middle, richer, and richest (highest 20%).

[b]The model did not converge.

[c]Respondents' education level was classified as no education (0 years of schooling), primary education (1–5 years of schooling), secondary education (6–12 years of schooling), and higher education (13 or more years of schooling).

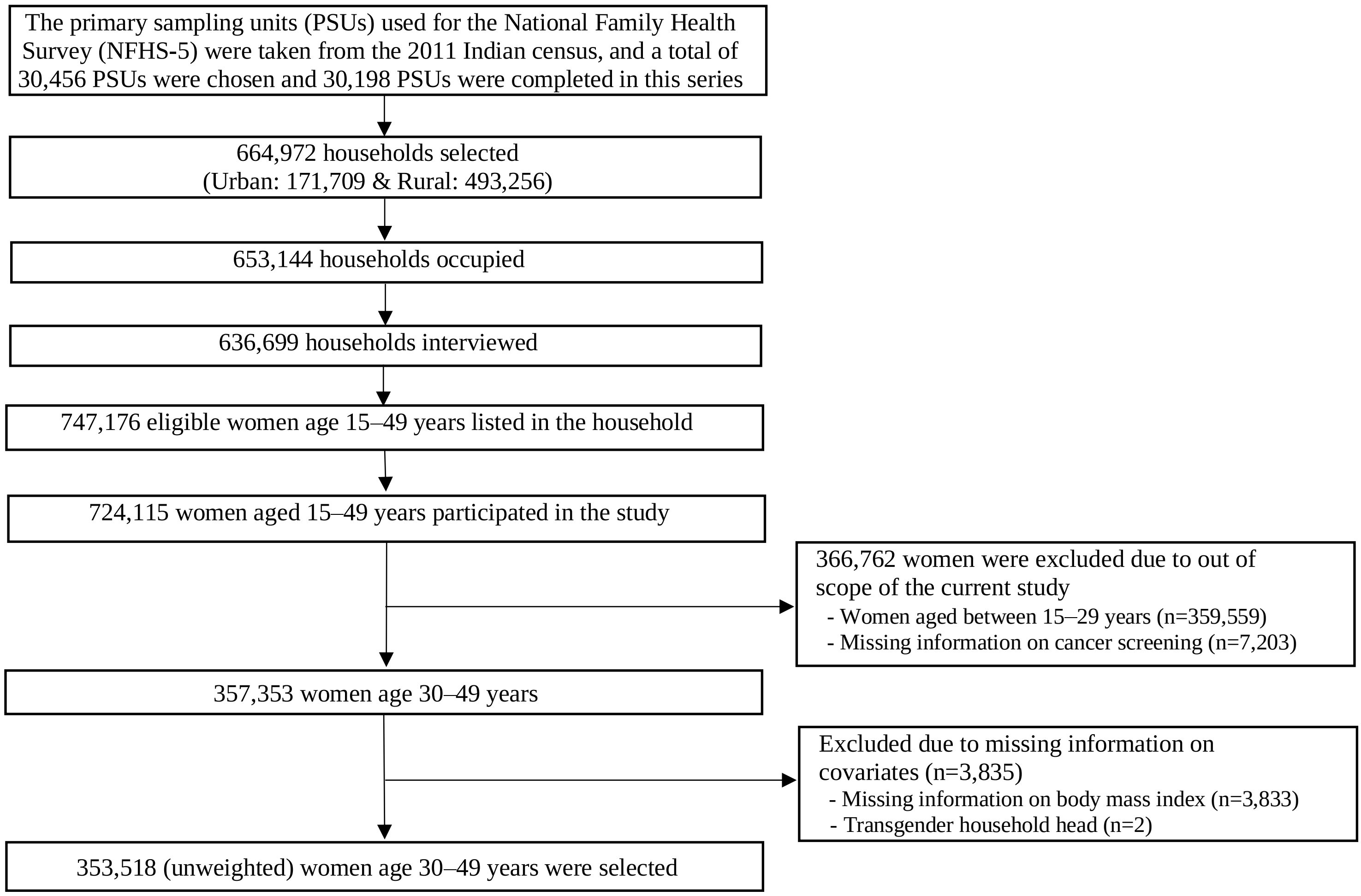

eFigure 1. Participant selection flowchart, the 2019-21 National Family Health Survey conducted in India

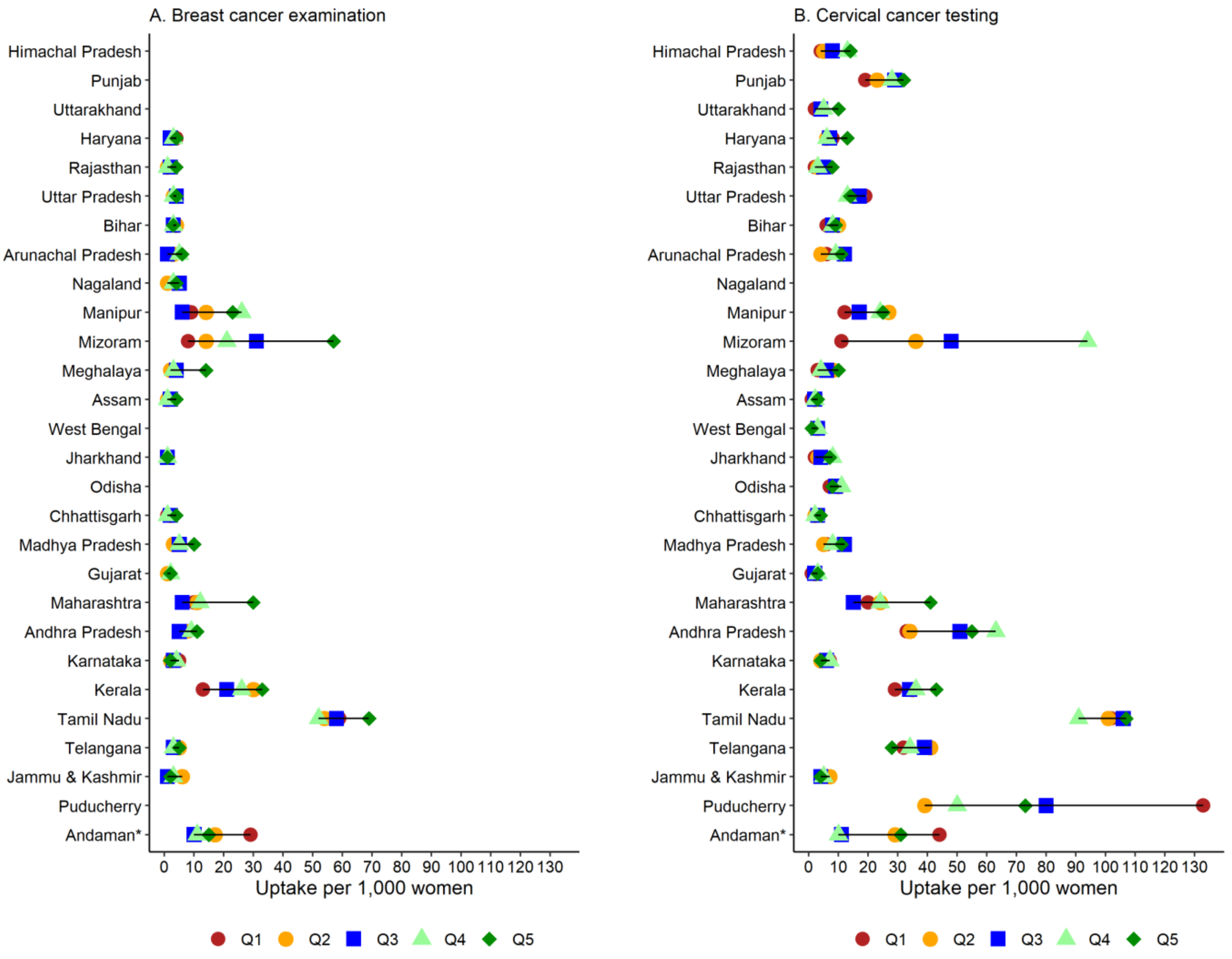

eFigure 2. Uptake of breast cancer examination and cervical cancer test among Indian women by socioeconomic status across states and union territories. *Andaman & Nicobar Islands. Panel A presents the uptake of breast cancer examination by household socio-economic status and panel B presents the uptake of cervical cancer testing by household socio-economic status. Q1, poorest quintile; Q2, poorer quintile; Q3, middle quintile; Q4, richer quintile; Q5, richest quintile. Exact values are presented in eTable 5. Household socioeconomic status was measured using the wealth scores. The households were ranked based on these scores and subsequently divided into quintiles, each representing 20% of the population. These quintiles were categorized as follows: poorest (lowest 20%), poorer, middle, richer, and richest (highest 20%).

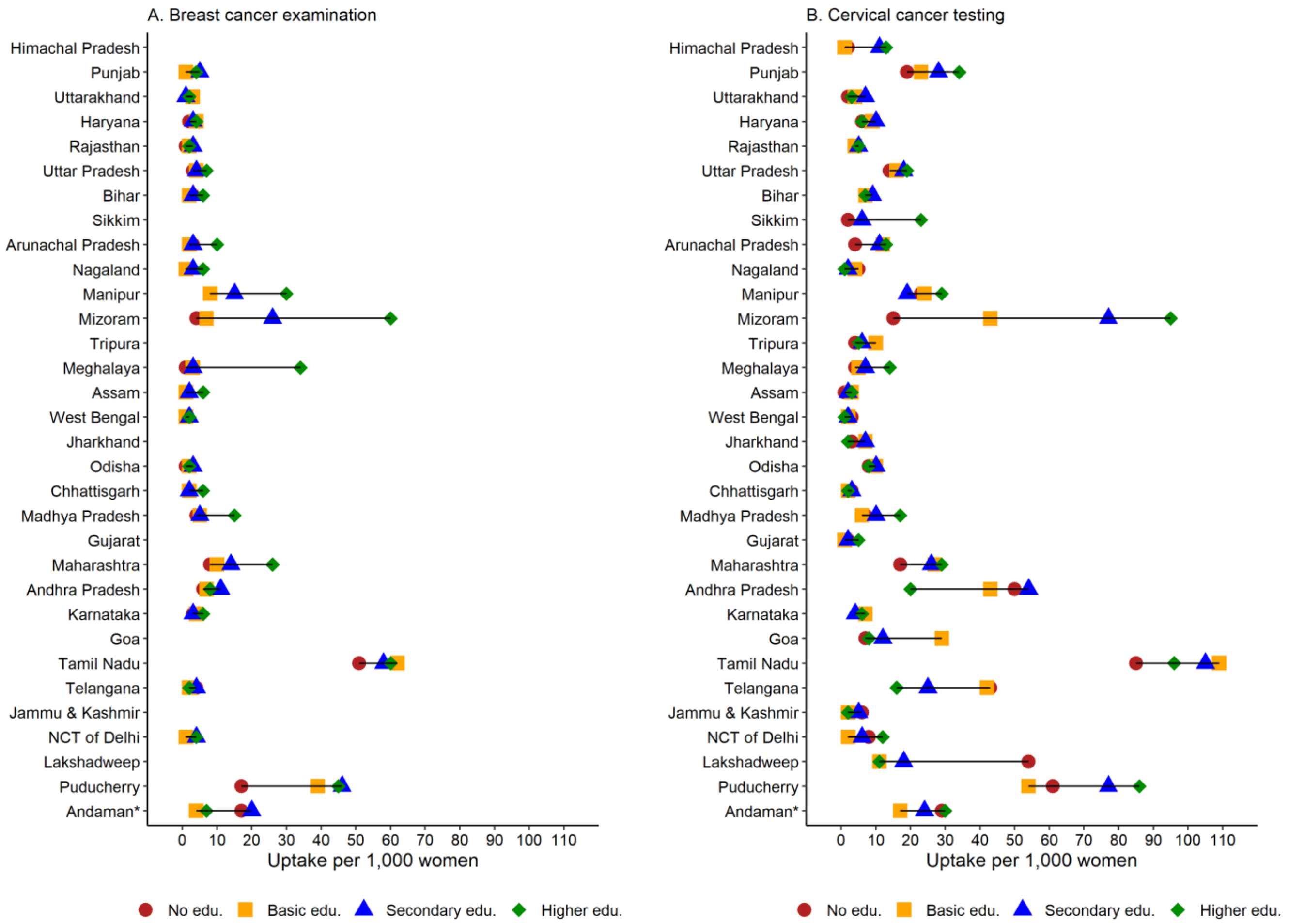

eFigure 3. Uptake of breast cancer examination and cervical cancer test among Indian women by educational status across states and union territories. NCT of Delhi, National Capital Territory of Delhi; *Andaman & Nicobar Islands. Panel A presents the uptake of breast cancer examination by women's level of education and panel B presents the uptake of cervical cancer test by women's level of education. Exact values are presented in eTable 6. Respondents' education level was classified as no education (0 years of schooling), primary education (1–5 years of schooling), secondary education (6–12 years of schooling), and higher education (13 or more years of schooling).

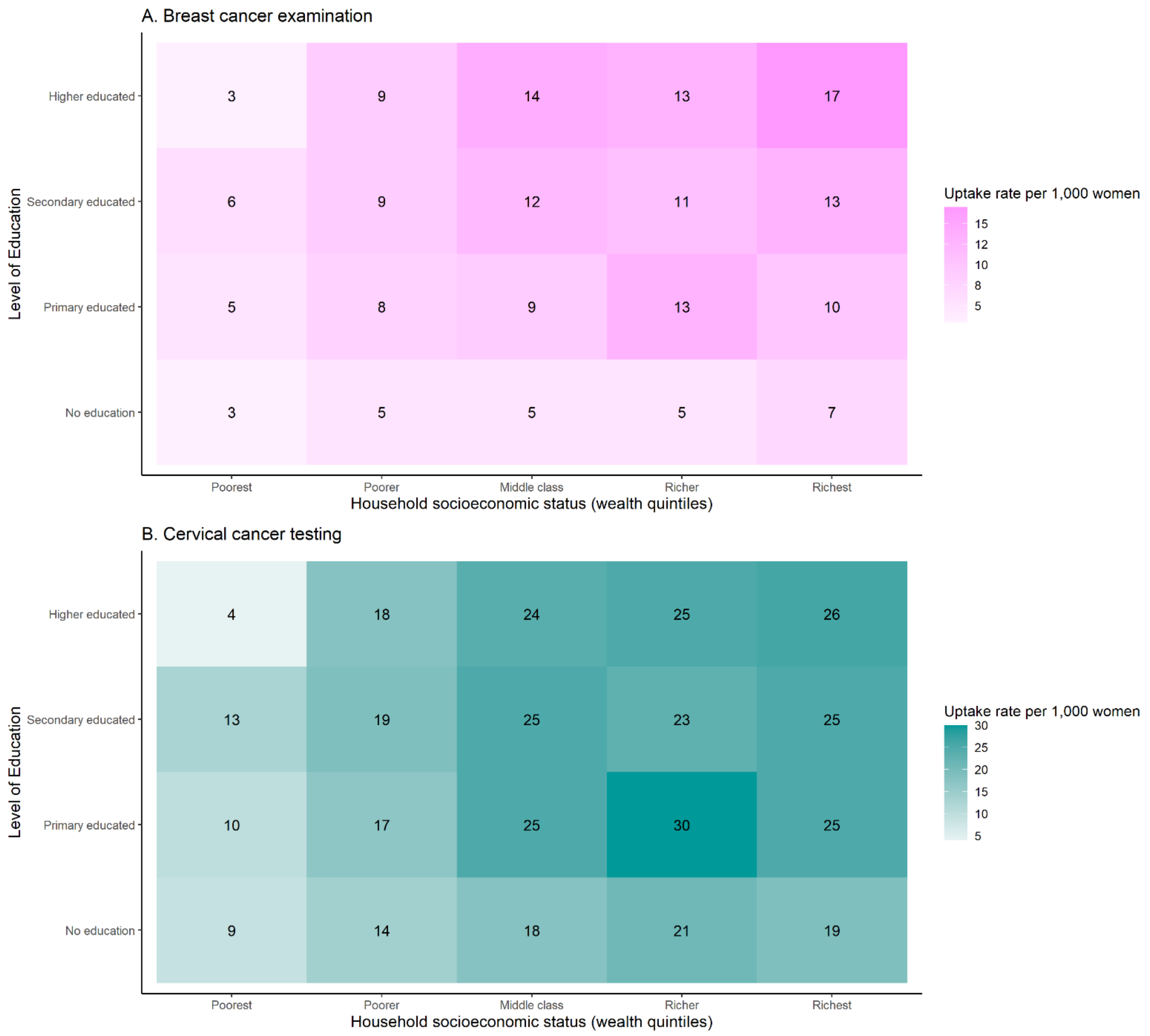

eFigure 4. Heatmap for the uptake of breast examination and cervical cancer test among Indian women (combination of socioeconomic status and education) at the national level. Panel A presents the uptake of breast cancer examination across different levels of women's education and household socio-economic status. Panel B presents the uptake of cervical cancer test by women's level of education and household socio-economic status. Respondents' education level was classified as no education (0 years of schooling), primary education (1–5 years of schooling), secondary education (6–12 years of schooling), and higher education (13 or more years of schooling). Household socioeconomic status was measured using the wealth scores. The households were ranked based on these scores and subsequently divided into quintiles, each representing 20% of the population. These quintiles were categorized as follows: poorest (lowest 20%), poorer, middle, richer, and richest (highest 20%).
